# *Pleurotus ostreatus *L-asparaginase’s use in food safety and biotechnology: from processing assistance to bioactive agent

**DOI:** 10.1186/s12934-026-03007-9

**Published:** 2026-05-04

**Authors:** Yehia A.-G. Mahmoud, Mohamed Bedaiwy, Maha M. Salem, Samar Shamla, Omyma A. Awadallah

**Affiliations:** 1 Botany and Microbiology Department, Faculty of Science, Tanta University, Tanta, 31257 Egypt; 2 Biochemistry Division, Chemistry Department, Faculty of Science, Tanta University, Tanta, 31257 Egypt

**Keywords:** L-ASNase, *P. ostreatus* AUMC 16015, SSF, Biomedical and food applications

## Abstract

**Background and aim:**

L-ASNase has attracted attention in many biomedical and food safety applications. Therefore, this study was designed to identify a novel and promising candidate for the sustainable biosynthesis of extracellular L-ASNase from *P. ostreatus* AUMC 16015 grown on various agricultural substrates under solid-state fermentation (SSF). Also, the enzyme’s wide-ranging bioactivities were examined, involving its antioxidant, anti-inflammatory, and antitumor properties, while evaluating its potential applications in food processing.

**Results:**

Optimal *P. ostreatus* AUMC 16015 L-ASNase production was 56.47 U/mL, which was attained under SSF conditions where the enzyme yield increased by 2.46-fold compared to pre-optimization conditions. Enzyme high purity was validated by a single distinct band at approximately 48 kDa on both SDS-PAGE and native PAGE analyses. The enzyme demonstrated high substrate specificity (*K*_*m*_ = 7.7 mM; *V*_*max*_ = 167.78 U/mL). Functionally, it exhibited strong antioxidant activity (2,2-diphenyl-1-picrylhydrazyl) (DPPH) IC_50_ = 48.28 µg/mL) and a robust anti-hemolytic effect (95.9% at 1000 µg/mL). L-ASNase exhibited its most potent inhibitory effect against Caco-2 cells at an IC_50_ of 5.49 ± 0.03 µg/mL, followed by MCF-7, which showed a slightly higher IC_50_ of 5.86 ± 0.08 µg/mL. Furthermore, L-ASNase significantly mitigated potato chips acrylamide formation, achieving a 9.6-fold decrease after 120 min of treatment. Additionally, Gas chromatography-mass spectrometry **(**GC-MS) showed that the potato’s chemical profile was significantly changed by L-ASNase treatment, with the introduction of numerous bioactive substances and the elimination of some potentially dangerous components.

**Conclusion:**

The biochemical activity of the purified L-ASNase suggested potential biomedical and food applications. This study is a trial for cost-effective enzyme production and supports a circular bioeconomy by converting waste into useful bioproducts. Future work should focus on scaling up production and testing its effects in living organisms to unlock this enzyme’s full commercial and medical potential.

**Graphical abstract:**

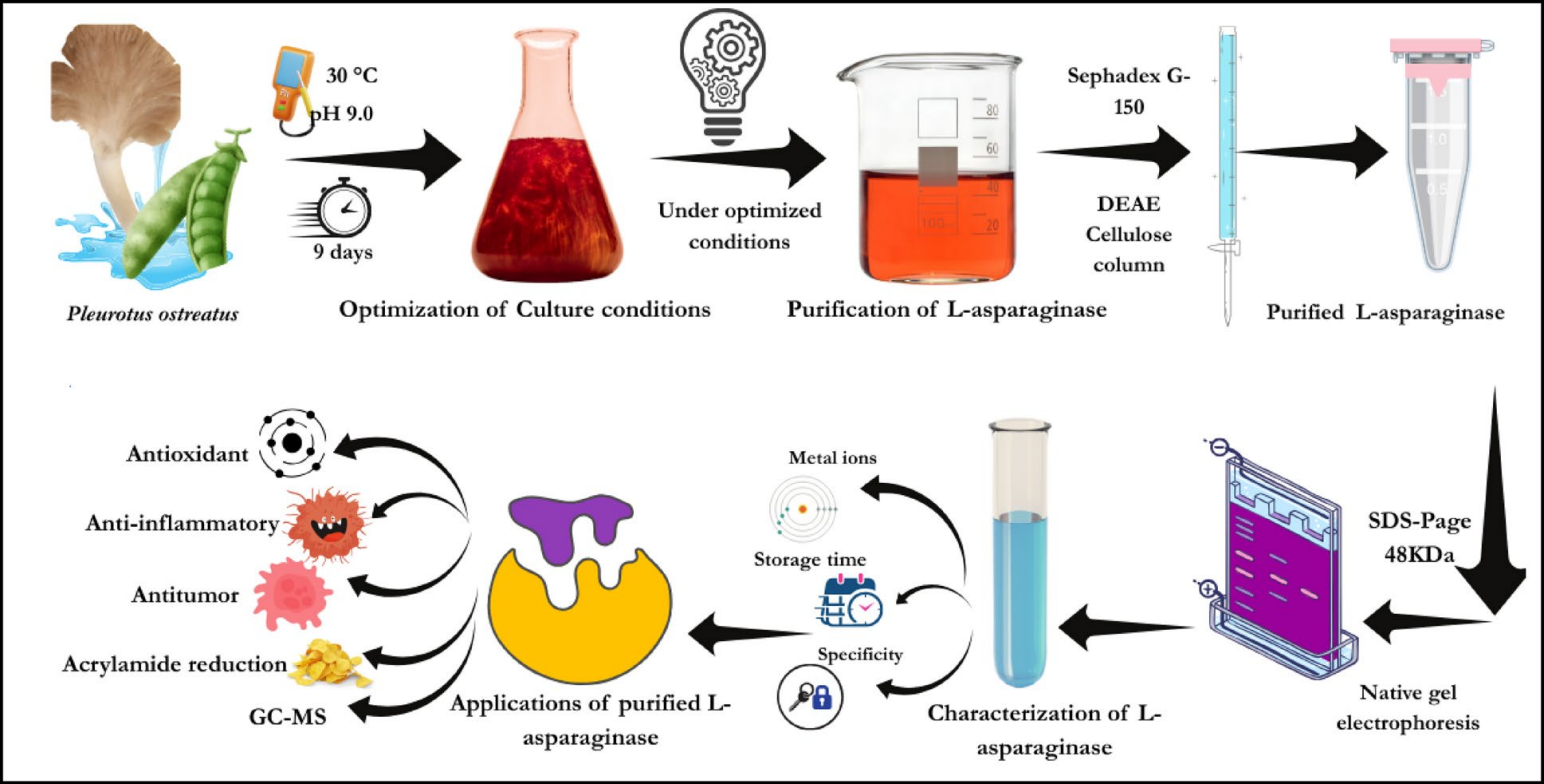

## Introduction

L-ASNase is distributed across animals, plants, the serum of specific rodent species, and microbes like bacteria, actinomycetes, yeasts, and filamentous fungi. Human L-ASNase-like enzymes have also been found in recent research, exhibiting biochemical activity that may have therapeutic and clinical value [1]. Since its initial identification in *Escherichia coli*, it has been established that microbes are the predominant origin of L-ASNase due to their ease of culture on a low-cost substrate, simplicity in optimizing the culture conditions for enzyme production, ease of genetic modification to boost yield, ability to produce the enzyme in large quantities, economical extraction and purification, and superior stability and consistency compared to plant and animal enzymes. Microorganisms are known to possess distinct asparaginase isoforms that vary in their subcellular localizations and biochemical properties, including periplasmic, intracellular, extracellular, and glutaminase-associated asparaginase variants, each contributing a specific functional role in basic metabolism in bacteria and fungi [2, 3]. The bacterial origin and glutaminase co-activity of clinically utilized L-ASNase preparations, which are produced from *E. coli* and *Erwinia* spp., are associated with hypersensitivity and other immune-related toxicities. In contrast, many filamentous fungi release extracellular L-ASNases, which facilitate easier purification and separation, while also exhibiting favorable biochemical properties, including a broader pH stability range, higher thermal stability, lower glutaminase co-activity, and improved catalytic efficiency. These characteristics contribute to reduced toxicity and enhanced therapeutic potential, and potent anticancer and acrylamide-mitigating effects. Consequently, filamentous fungi are being investigated as alternative sources of L-ASNase. Even though in vitro and in silico data indicate that certain fungal L-ASNases may have a more favorable immunological profile than bacterial enzymes, their glycosylation patterns and antigenicity are still poorly understood and need to be thoroughly assessed in preclinical and clinical studies before any conclusive claims regarding decreased toxicity can be made [4, 5].

Furthermore, by using affinity tags and tailored secretion signals, recombinant expression systems like *E. coli* and *Pichia pastoris* have significantly increased the scalability and simplicity of L-ASNase purification. High-yield extracellular synthesis is made possible by these yeast-based systems, which also present the possibility of engineering glycosylation patterns to improve protein stability and lower immunological responses [6, 7].

Since excessive glutamine depletion greatly increases toxicity without significantly improving antileukemic efficacy, low glutaminase co-activity has emerged as a crucial criterion in the construction of safer, more selective therapeutic L-ASNase. The development of engineered or alternative-source enzymes with low glutaminase activity that still effectively deplete asparagine in leukemic cells has been spurred by clinical and preclinical research linking high glutaminase activity of *E. coli* and *Erwinia* L-ASNase to hepatic dysfunction, pancreatitis, coagulation abnormalities, and neurotoxicity [8, 9].

Nearly all industrial-scale synthesis of L-ASNase involves microbial submerged fermentation (SmF). Among the many drawbacks associated with this approach are the low concentration of product creation and the ensuing need to handle, reduce, and dispose of vast amounts of water during downstream operations. As a result, the SmF technique is expensive, highly challenging, and represents an inadequately understood unit operation. In contrast, solid-state fermentation (SSF) is an alternate approach with many benefits [10]. These benefits encompass reduced energy consumption, easier downstream processing with reduced catabolic repression, elevated volumetric productivity, increased product concentration, satisfactory product stability, and minimal mechanical complexity, collectively contributing to lower overall energy demands [11]. SSF uses a simple and cost-effective medium because its primary substrates are widely available and reasonably priced industrial and agricultural wastes [12]. Therapeutic enzymes are used to treat illnesses like cancer, inflammation, and severe conditions such as autism, multiple sclerosis, and chronic lung disease. An amide hydrolase enzyme called L-ASNase (E.C. 3.5.1.1) catalyzes the deamidation of L-asparagine to L-aspartic acid and ammonia, as well as the conversion of L-glutamine into L-glutamic acid and ammonia. This enzymatic action underlies its use as a chemotherapeutic agent. The therapeutic rationale for L-ASNase relies on the fact that tumor cells possess insufficient levels of L-asparagine synthetase (EC 6.3.5.4) limits their capacity to biosynthesize L-asparagine, an amino acid that is typically non-essential [13]. Because these malignant leukocytes lack this enzyme, which impairs cellular processes and leads to cell death, they rely on this amino acid from external sources for maintenance and rapid growth. Therefore, when L-ASNase is present, the tumor cells lose a crucial growth factor and may not survive [14]. The action of L-ASNase decreases the accessible free exogenous concentration of L-asparagine. Crucially, this treatment induces a state of terminal starvation specifically in vulnerable tumor cells, but it does not affect the function of normal cells, which can synthesize enough for their needs [15]. The therapeutic effectiveness of L-ASNase depends on various factors, including the enzyme’s source and hydrolysis rate to its bioprocessing grade. The development of anti-asparaginase antibodies and tumor cell resistance further limits its effectiveness in a therapeutic environment [3].

The goal of recent developments has been to improve L-ASNase therapy through pharmacokinetic optimization, toxicity reduction, and enzyme stability. High-yield secretion and easier purification are made possible by recombinant expression in hosts like *E. coli* and *P. pastoris*, and protein engineering methods like site-directed mutagenesis, PEGylation, and the creation of low glutaminase variants have produced enzymes with longer half-lives, lower immunogenicity, and preserved anticancer efficacy. Together, these advancements aid in the creation of safer and more potent next-generation L-ASNase therapies [16].

Furthermore, L-ASNase’s therapeutic bioactivity includes an anti-inflammatory action. Inflammation constitutes a component of the intricate response of the body’s vascular tissues to harmful stimuli, including infections, allergens, or damaged cells [17]. Numerous signaling mediators produced by mast cells, macrophages, granulocytes, lymphocytes, platelets, and complement activation factors mediate the inflammatory response [18]. In addition to their use in medicine, L-ASNases are identified as effective acrylamide inhibitors in thermally processed foods. Acrylamide was classified as a carcinogen and a strong neurotoxin by the International Agency for Research on Cancer. Acrylamide levels are often successfully mitigated by more than 80% by enzymatic treatment using microbial and fungal L-ASNases without sacrificing the food’s flavor. This environmentally friendly enzymatic method has been successfully used in a variety of food products, including coffee, potatoes, and cereals, demonstrating its potential for industrial-scale food safety interventions. The scalability and regulatory acceptance of L-ASNase therapies as a crucial tactic to reduce dietary acrylamide exposure and improve public health protection is supported by ongoing research [19, 20].

The principal aim of this investigation was to enhance the biosynthesis of *P. ostreatus* L-ASNase via SSF on agricultural wastes. Growth parameters were systematically optimized to maximize enzyme yield, followed by purification and comprehensive biochemical characterization. Additionally, the enzyme’s potential antioxidant, antileukemic, and anti-inflammatory activities were evaluated. The study also investigated L-ASNase efficacy in inhibiting acrylamide production in starchy food applications.

## Materials and methods

### Chemicals

L-asparagine 99% (CAS# 5794-13−8), NaH_2_PO_4_.H_2_O 99% (CAS# 10049-21−5) Na_2_HPO_4_.H_2_O 99% (CAS# 10140-65−5), Tris-HCl (CAS# 1185-53−1), Trichloroacetic acid (TAC) 99% (CAS# 76-03−9; cat#489), Coomassie Brilliant Blue G-250 (CAS# 6104-58−1), Phenol red 99% (4-[3-(4-hydroxyphenyl)−1,1-dioxo-2,1lambda6-benzoxathiol-3-yl] phenol) (CAS# 143-74−8), KOH 85% (CAS# 1310-47−3), KI (CAS# 7681-11−0), HgCl_2_ 99% (CAS# 7487-94−7), Ammonium sulphate (CAS# 7783-20−2), L-glutamine (CAS# −9), L-aspartic acid (CAS# 56–84−8), Acetamide (CAS# 1449-72−5), Glycine (CAS# 56-40−6), DPPH (2,2-diphenyl-1-picrylhydrazyl) (CAS# 1898-66−4), Indomethacin (CAS# 53–86−1), MMT (3-[4,5-dimethylthiazol-2-yl−2,5 diphenyl tetrazolium bromide) (CAS# 298-93−1), Acrylamide (CAS# 97-06−1). The high-grade analytical reagent chemicals used in this study were procured from Sigma-Aldrich (Egypt).

### Organism collection

A mushroom was collected from the Delta region (23/10/2021, autumn season), growing on a decaying hardwood, Egypt. A pure culture of the mushroom isolate was obtained from fruiting body which was surface sterilized with 3% chlorine for five minutes. The fruiting bodies were divided in half lengthwise, and small pieces (0.5 × 0.5 cm) were excised from the junction between the cap and the stem. These pieces were placed on sterile petri dishes containing sterilized Potato Dextrose agar (PDA) medium to which 3 ppm benomyl and 10 ppm aureomycin were added to prevent the development of fungal and bacterial contaminations respectively. The plates were incubated at 25 °C to monitor mycelial growth. Samples exhibiting homogeneous mycelium without contamination were selected [21, 22].

### Morphological description

The isolated mushroom was determined by the morphology and color of the fruiting bodies, sporulation, gill-to-stalk connection, and basidiospore shapes, and the taxonomic keys [23], it was concluded to be *P. ostreatus*.

### Molecular identification of*P. ostreatus* using the 18 S rRNA

Molecular identification of *P. ostreatus* was carried out to confirm the identification. *P. ostreatus* was incubated at 25 °C on Czapek’s Yeast Extract Agar (CYA) for five days. After being aseptically scraped and suspended in 100 µl of sterile distilled water, a tiny portion of fungal mycelia was cooked for 15 min and allowed to cool before being stored at −70 °C. Using universal ITS1 and ITS4 primers, the ITS region of nuclear rDNA was amplified by PCR. After being purified and verified using agarose gel electrophoresis, amplicons were sequenced and verified by agarose gel electrophoresis, and both directions of sequencing were performed. ITS sequences were examined using BLAST in comparison to NCBI databases, and phylogenetic alignment was performed with closely related GenBank entries [24].

### Maintenance of*P. ostreatus*

*P. ostreatus* was subcultured and preserved on PDA medium. After a 7-day incubation period at 28 °C, the plates and slants were transferred to 4 °C for storage.

### Agricultural wastes

Ten different types of agricultural waste, namely; wheat straw, sugarcane bagasse, rice straw, soybean husks, cottonseed oil cake, pea peel, wheat bran, potato peel, corn seeds, and apple peel, were collected from agricultural farms located in Gharbia Governorate, Egypt. The tested wastes were assessed separately for their suitability as SSF substrates for L-ASNase production. Each substrate was washed with distilled water to remove impurities and sun-dried for 3 days to reduce the moisture content. The dry materials were then mechanically ground separately and sieved to a uniform particle size of 3 mm before use in SSF. The prepared agricultural wastes served as the sole carbon source and solid support matrix for the SSF process.

### Qualitative screening of L-ASNase using a rapid plate assay

*P. ostreatus* was assessed for L-ASNase production, and the rapid plate assay was conducted following the protocol established by Gulati et al. [25]. Under aseptic conditions, modified Czapek-Dox agar medium was prepared and incorporated with the only source of nitrogen, L-asparagine in the medium (100 mL), supplemented with a volume of 0.3 mL of phenol red coloring at a concentration of 0.005 g/L prepared in ethanol for pH indication, and the pH was modified to 6.5. After autoclaving, the medium was dispensed into 9 cm petri dishes, where it was left to harden, then inoculated separately with *P. ostreatus* (5 mm disc). For control plates, uninoculated plates served as a negative control, and inoculated plates served as a positive control (Sodium nitrate was substituted for asparagine in the medium). All plates were incubated at 25–28 °C for 72 h. Following incubation, the colony diameters (cm) of *P. ostreatus* and the corresponding pink zones that appeared around the colony were measured.

### L-ASNase detection in both extracellular or intracellular extracts of*P. ostreatus*

Autoclaved modified Czapek-Dox broth medium (50 mL) was added to each of the 250 mL Erlenmeyer flasks (L-asparagine 10.0, KH_2_PO_4_ 1.52, KCl 0.52, MgSO_4_.7H_2_O 0.52, ZnSO_4_.7H_2_O trace, CuNO_3_.3H_2_O trace and FeSO_4_.7H_2_O trace g/L), were inoculated separately with 3 discs (each 5 mm) of seven days old culture of *P. ostreatus* and were cultured at 28 °C with agitation at 150 rpm for a duration of seven days using a rotary shaker. After the incubation time, the Whatman No. 1 qualitative filter paper was used to filter the broth culture. The culture filtrate of each flask was centrifuged at 4 °C for 20 min using a cooling centrifuge at 6,000 rpm [26]. The produced supernatant was referred to as a crude enzyme and was employed for the quantitative determination of extracellular L-ASNase catalytic efficiency, measured spectrophotometrically at 450 nm. While the mycelial biomass of *P. ostreatus* was subjected to cell lysis and subsequently analyzed to determine intracellular L-ASNase activity, the mycelium left over from the filtration process was rinsed twice with a pH of 8.6, 50 mM Tris-HCl, and then resuspended in the identical lysis buffer to get crude cell lysate. The fungal mycelial mass was subsequently disrupted via sonication using a probe sonicator over three 15-s periods, with an interval of 45 s between periods under cooling conditions at 4 °C. Following centrifugation at 4 °C for 15 min at 6,000 rpm, cellular debris and intact cells were eliminated by filtration, and the supernatant was collected to measure intracellular enzyme activity using a spectrophotometer at 450 nm [27].

### Effect of agricultural wastes on*P. ostreatus* L-ASNase production (SSF)

Under aseptic conditions, five grams of each substrate were individually weighed into sterile three 250 mL Erlenmeyer flasks as replicates, for each substrate, and all flasks were moistened separately with 4 mL distilled water (the desired moisture level) as described by Meghavarnam and Janakiraman [28]. The contents of each flask were thoroughly and carefully mixed, and the flasks were autoclaved at 121 °C under 1.5 atmospheric pressure for 15 min and cooled well to room temperature before use. Three mycelial plugs (5 mm) of *P. ostreatus* were used to inoculate each flask from a 7-day-old culture and incubated at 28 °C for 7 days. After incubation, the enzyme extraction from each inoculated substrate was carried out, then mixed separately with 25 mL of 0.1 M sodium phosphate buffer (pH 8.0) in each flask containing the crude L-ASNase and kept on a rotary shaker at 150 rpm and 28 °C for 1 h. Then, the mixtures were subsequently filtered through sterile cotton cloth, and the resulting filtrates were subjected to centrifugation at 6,000 rpm for 20 min at 4 °C. The supernatant obtained from each used substrate was analyzed separately for enzyme activities [29].

### L-ASNase enzyme activity assay

L-ASNase activity was measured quantitatively by Nesslerization [30], which determines the rate of L-asparagine hydrolysis by quantifying liberated ammonia. In triplicate, 0.5 mL of 0.01 M L-asparagine was mixed with 0.5 mL of 0.05 M Tris-HCl buffer (pH 8.6), 0.5 mL of the isolated enzyme, and 0.5 mL of distilled water to a final volume of 2.0 mL. The reaction mixture was incubated at 37 °C for 30 min, then terminated by adding 0.5 mL of 1.5 M trichloroacetic acid (TCA). Subsequently, 0.1 mL of this mixture was diluted with 3.7 mL of distilled water, followed by the addition of 0.2 mL Nessler’s reagent and incubation at 37 °C for 15–20 min. Absorbance was measured at 450 nm with the spectrophotometric analyzer. The heat-inactivated enzyme served as a control. Enzyme activity was reported in International Units (IU), defined as the amount of enzyme that catalyzes the formation of 1 µmol of ammonia per min per mL.

### Optimization of culture conditions to maximize*P. ostreatus* L-ASNase under SSF

Following initial screening of the ten agricultural wastes, the waste supporting the highest L-ASNase production was selected as the sole solid substrate for subsequent optimization studies. Culture conditions were then optimized to identify factors maximizing L-ASNase biosynthesis under SSF. The optimal condition achieved by any factor was fixed for subsequent experiments. The fermentation parameters were; pretreatment of pea peel with different conditions (hot sterilized distilled water at 70 °C and cold sterilized distilled water), moistened of pea peel with freshly prepared modified Czapek-Dox broth medium (pH 7.0) or with sterilized distilled water, particle sizes were 2, 3, 4 mm, fine, intermediate, and coarse, respectively, the weight of the substrate (3, 5, 7, 9, and 11 g), initial substrate moisture content (1:1, 1:2, 1:3, 1:4, 1:5, 1:6, 1:7 g/mL), incubation period (4, 7, 9, 11, 13, and 15 days), inoculum size (3, 5, 7, 9, 11, and 13 mycelial plugs (5 mm)), inoculum age (7, 14, 21, and 28 days), incubation temperature (25, 28, 30, 35 and 40 °C) and initial pH (5, 6, 7, 8, 9, 10, and 11) adjusted with 1 N HCl or 1 N NaOH), on L-ASNase production were systematically investigated [31, 32]. For each experiment, all other parameters were kept at their optimal level. L-ASNase activity was quantitatively assayed, with all experiments performed in triplicate for each factor as mentioned before.

## Purification of L-ASNase

### Ammonium sulphate precipitation

The crude isolated enzyme was subjected to ammonium sulfate fractionation, where L-ASNase activity was obtained at 80% to achieve the first stage of enzyme purification [33]. Then, the precipitate was collected and subsequently re-suspended in a minimal volume of Tris-HCl buffer (0.05 M at pH 8.6), dialyzed, and the enzyme activity was measured as described before.

### Sephadex G-150 column chromatography

Gel filtration chromatography was used to purify the dialyzed ammonium sulfate fraction on a Sephadex G-150 column as described by [34]. After swelling the Sephadex G-150 resin for 2 days, it was packed into a column measuring 23 × 1.2 cm and equilibrated with two column volumes of 0.05 M Tris-HCl buffer, adjusted to pH 8.6. Elution was performed at a flow rate of 2 mL per 5 min. Protein was measured at 280 nm and enzymatic activity were assayed in each collected fraction. Then, fractions exhibiting elevated enzymatic activity were combined and preserved at 4 °C for subsequent purification via ion-exchange chromatography.

### Ion exchange chromatography

L-ASNase-containing precipitate obtained from the Sephadex G-150 column was further purified using a prepacked DEAE cellulose ion exchange column, a weak anion exchanger. Column equilibration was performed using 10 column volumes of 0.05 M Tris-HCl buffer (pH 8.6). The sample was loaded onto the top of the chromatography column and eluted with a linear sodium chloride gradient between 0.05 to 0.3 M. Eluted fractions were collected at a rate of 3 mL per 5-min interval. The fractions were analyzed to determine both protein absorbance at 280 nm and enzyme activity, and the most potent fractions exhibiting the maximal activity were combined and subsequently analyzed by SDS-PAGE [35].

### Estimation of L-ASNase molecular weight

SDS-PAGE was employed to determine the approximate molecular weight of pure L-ASNase according to the method of Laemmli [36]; using 10% (w/v) gels developed at room temperature. Coomassie Brilliant Blue R-250 staining was used to visualize protein bands and compared to a pre-stained protein marker ladder (BLUeye Prestained Protein Ladder, 10–245 kDa; Cat# PM007-0500).

### Native gel electrophoresis

Native PAGE, or non-denaturing PAGE, was performed similarly to SDS-PAGE, using Tris-HCl buffer (375 mM at pH 8.8) but omitting SDS as described by Warangkar and Khobragade [37]. Briefly, the L-ASNase fraction, purified via DEAE cellulose chromatography, was loaded onto a Tris-HCl polyacrylamide gel without SDS and electrophoresed at 4 °C. After a 2 h staining period with 0.1% Coomassie Brilliant Blue R-250, the gel was destained, and protein bands were visualized and analyzed using a Gel Documentation System software. The intact L-ASNase molecular weight was estimated by comparing its band migration to that of standard native molecular weight markers.

### Characterization of the purified L-ASNase

####  Effect of pH on L-ASNase activity and stability

The pH effect on the purified enzyme was investigated across a pH range from 3.6 to 10.6 employing a series of 0.05 M buffers to test various pH values as acetate buffer (pH 3.6, 4.4, and 5.6), phosphate buffer (pH 6.2, 7, and 8), and carbonate buffer (pH 9.2, 10, and 10.6). The enzyme’s pH stability was assessed by pre-incubating it with these previous pH values at different time intervals (5, 10, 15, 20, and 24 h) at 4 °C. The activities were determined under standard assay conditions [38].

#### Effect of temperatures on L-ASNase activity and stability

The thermal tolerance of purified L-ASNase was examined across a temperature spectrum ranging from 20 to 70 °C. Additionally, the enzyme’s thermal stability was quantified by measuring residual activity following the methodology established by [39] and the enzyme was pre-incubated for varying durations, ranging from 10 to 60 min, before further analysis at the previously mentioned various temperatures. Then, the enzyme activity was measured under standard assay conditions.

####  Effect of different compounds and surfactants on L-ASNase activity

The impacts of different compounds and surfactants, SDS, Dimethyl sulfoxide (DMSO), Urea, Tween 80, Ethylene diamine tetra acetic acid (EDTA), and Triton X-100, at 1 mM, were investigated. They were pre-incubated with purified L-ASNase for 30 min before assaying the enzyme hydrolytic activity. In the absence of these different compounds and surfactants, the enzyme’s activity was assumed to be 100%, serving as the control [40].

#### Effect of different metal ions

Effects of several mono- and divalent cations (Mg^+ 2^, Cu^+ 2^, Hg^+ 2^, K^+^, Zn^+ 2^, Fe^+ 2^, Na^+^, and Ca^+ 2,^ at concentrations 1, 5 and 10 mM) on the biocatalytic efficiency of purified L-ASNase were evaluated. In brief, for 1 h, pre-incubation of L-ASNase was conducted with metal ion solutions at varying concentrations. The enzyme’s activity was 100% when no metal ions were added, and this was used as the control [41].

#### Effect of substrate specificity

The substrate specificity of pure L-ASNase was examined using glycine, acetamide, L-glutamine, L-glutamic acid, and L-asparagine, each at a 0.01 M concentration under standardized conditions. The experiment was conducted by substituting the relevant amino acid for 0.01 M L-asparagine (control), and the activity was ascertained as previously mentioned [42].

#### Effect of storage times at different temperatures

The relative activity 100% was determined to be the activity of the stock solution at the zero-time point (t_0_). After that, some aliquots of the stock solution were stored at two different conservation temperatures in sterile 1.5 mL microtubes at 4 °C and −20 °C. After rewarming to 37 °C for every storage time, pure L-ASNase residual activity was then assessed every week from (1–6) at 4 °C and every month from (1–8) at 20 °C [43].

### Kinetic parameters

The pure L-ASNase’s kinetic characteristics were evaluated by measuring its catalytic activity across different L-asparagine substrate concentrations. Linear regression of Lineweaver-Burk plots was employed to determine the kinetic parameters *K*_*m*_ and *V*_*max*_ based on the Michaelis-Menten equation [41].

## Biological activity of the purified L-ASNase

### Therapeutic application (*in vitro*)

#### Antioxidant activity

##### DPPH radical scavenging

The samples were assayed for radical scavenging activity using the DPPH method, as described by Brand-Williams et al. [44]. A 3 mL aliquot of a 0.024% (w/v) working DPPH solution was mixed with 100 µL of the purified L-ASNase obtained from previously fractionated column aliquots (fractions F56 to F60), and the mixture was incubated in the dark for 1 h. Subsequently, the fraction demonstrating the highest enzymatic activity was subjected to serial dilution and assayed by the same DPPH method. Absorbance readings for the mixtures were recorded at 517 nm to calculate the percentage scavenging activity, applying the formula that follows:

1 $${\text{DPPH scavenging }}\left( \% \right){\text{ }} = {\text{ }}\left[ {{\mathrm{(A}}0{\text{ }} - {\text{ A1}}} \right)/{\mathrm{A}}0]{\mkern 1mu} {\mkern 1mu} \times {\mathrm{1}}00 $$

A0: The control absorbance, and A1: represents the DPPH absorbance after reacting with the purified L-ASNase. Ascorbic acid was a standard [45].

#### Anti-inflammatory activity

Anti-inflammatory activity of the obtained purified *P. ostreatus* L-ASNase was examined at the Regional Centre for Mycology and Biotechnology, Al-Azhar University, Cairo Governorate, Egypt, using the antihemolytic membrane stabilization assay. Briefly, 3 mL of Fresh whole blood was drawn from healthy volunteers and placed into heparinized collection tubes, followed by centrifugation at 3000 rpm for 10 min. Then the red blood cell (RBC) pellets were reconstituted in a volume of normal saline (v/v) equivalent to the supernatant that was recovered. With an isotonic sodium phosphate buffer (10 mM) adjusted to pH 7.4, a 40% (v/v) suspension of the resuspended erythrocyte pellets was obtained. The buffer was composed of 0.2 g NaH_2_PO_4_, 1.15 g Na_2_HPO_4_, and 9.0 g NaCl dissolved in 1 L of distilled water. The reconstituted resuspended pellets were utilized as follows. Furthermore, erythrocyte hemolysis induced by a hypotonic solution evaluated the membrane-stabilizing action of pure L-ASNase [46]. The pure L-ASNase sample was dissolved in distilled water, a hypotonic solution. Centrifuge tubes were filled with 5.0 mL of a hypotonic solution at different enzyme concentrations, ranging from 100 to 1000 µg/mL. 5 mL of an isotonic solution with (100–1000 µg/mL) of pure L-ASNase was also transferred into centrifuge tubes. The control tubes comprised 5.0 mL of purified water and 5.0 mL of indomethacin solution (200 µg/mL), respectively. After adding 0.1 mL of erythrocyte suspension to every tube, the contents were gently mixed and incubated at 37 °C for 1 h. The tubes were then centrifuged at 3000 rpm for 3 min. A Milton Roy spectrophotometer was used to quantify hemoglobin levels in the supernatant by recording absorbance at 540 nm. Using the modified technique outlined, the membrane stability or hemolysis inhibition percentage was quantified by Shinde et al. [46].

2 $${\text{Membrane stabilization }}\left( \% \right){\text{ }} = {\mathrm{1}} - \left[ {{\mathrm{OD2}} - {\mathrm{OD1}}/\left( {{\mathrm{OD3}} - {\mathrm{OD1}}} \right)} \right]\,\, \times {\mathrm{1}}00 $$

where OD₁ represents the test sample in isotonic solution, OD2 for the test sample in hypotonic solution, and OD3 for the control sample in hypotonic solution. The IC₅₀ value was determined as the sample concentration that resulted in 50% inhibition of RBC hemolysis under the assay conditions.

#### Anti-tumor activity

The National Cancer Institute in Cairo, Egypt, supplied six human tumor cell lines. These were grown as monolayers in Roswell Park Memorial Institute (RPMI-1640) medium, enriched with antibiotics (100 µg/mL of streptomycin and 100 µg/mL of penicillin), 2 mM glutamine, and 5% heat-inactivated fetal bovine serum (FBS). The cells were then incubated at 37 °C in a humidified environment with 5% CO_2_ [47, 48]. Using MTT assay (3-(4,5-dimethylthiazol)−2,5-diphenyltetrazolium bromide), in vitro cytotoxic impact of pure L-ASNase enzyme was prepared at different concentrations for testing: 50, 25, 12.5, 6.25, 3.125, and 1.562 µg/mL were investigated on the growth of panel tumor cell lines which were Hepatocellular carcinoma cell line (HepG-2), Breast carcinoma cell line (MCF-7), Colon carcinoma (Caco-2) Lung cells (A549), Acute Myoblastic leukemia (Kasumi-1), Histiocytic lymphoma (U937) and normal cells (WI-38). The relationship between cell viability and pure L-ASNase concentration was plotted to determine the effective concentration needed to prevent 50% of cell development (IC₅₀, µg/mL) graphically. The IC_50_ value represents the L-ASNase concentration caused the cell viability to drop by 50% in comparison to the control. Cell viability was determined using the formula:

3 $${\text{Variability percentage}} = ({\mathrm{ODt}} /{\mathrm{ODc}}) \times 100 $$

ODt represents the mean optical density of triplicate wells treated with each concentration of L-ASNase, and ODc is the mean optical density of untreated control cells. The half maximal inhibitory concentration (IC₅₀) was defined as the concentration of L-ASNase that caused a 50% inhibition of cell viability compared to the untreated control.

### Food processing aids

####  Determination of the effect of pure L-ASNase on acrylamide formation in fried potato

Fresh potatoes were cleaned, peeled, and sliced into 2-mm pieces. Slices were rinsed with deionized water to eliminate any remaining starch. After that, the potato slices were immersed in the enzyme solution exhibiting the highest activity, which was 60.88 IU/mL, at 37 °C for varying times (30, 60, 90, and 120 min) and dried on absorbent papers. After five min of cooking at 170 °C, the potato slices were allowed to cool to room temperature. Following cooling, 10 mL of deionized water and 2 g of fried chips were ultrasonically agitated for 30 min. Then, the mixture was spun at 6000 rpm in a centrifuge for 10 min. Using standard acrylamide, high-performance liquid chromatography (HPLC) was employed to ascertain the acrylamide mitigation level in both treated and untreated potato samples [49].

### GC-MS analysis

The chemical constitution of the two samples (control sample – treated sample with pure L-ASNase at 51.8 U/mL for 120 min) was analyzed using a Trace GC-1310-ISQ mass spectrometer equipped with a direct capillary column TG–5MS (30 m length ×0.25 mm internal diameter ×0.25 μm film thickness). The column oven temperature was initially set to 50 °C, then ramped up at 5 °C per min to 230 °C, and maintained for 2 min, before rising to the final temperature of 290 °C operating at 30 °C/min, where it was kept for 2 min. The mass spectrometer transfer line temperature was set at 260 °C, while the injector temperature was maintained at 250 °C. The carrier gas, helium, was utilized at a steady flow rate of 1 mL/min. A 3-min solvent delay was applied. An Autosampler AS1300 connected to the gas chromatograph was used to automatically inject diluted samples (1 µL) into split mode. Electron ionization (EI) was used to record mass spectra at 70 eV ionization energy spanning the 40–1000 mass-to-charge ratio (m/z) range while in full scan mode and with the ion source temperature set to 200 °C. Component identification was accomplished by comparing mass spectral data and retention durations to reference spectra from the NIST 11 and WILEY 09 libraries [50, 51].

### Statistical analysis

A total of three separate trials were conducted for the experiments, and the average results, along with the standard deviation (mean ± SD), are provided (*n* = 3). Statistical significance was assessed using one-way ANOVA.

## Results and discussion

### Qualitative screening of L-ASNase using a rapid plate assay

**Fig. 1:**
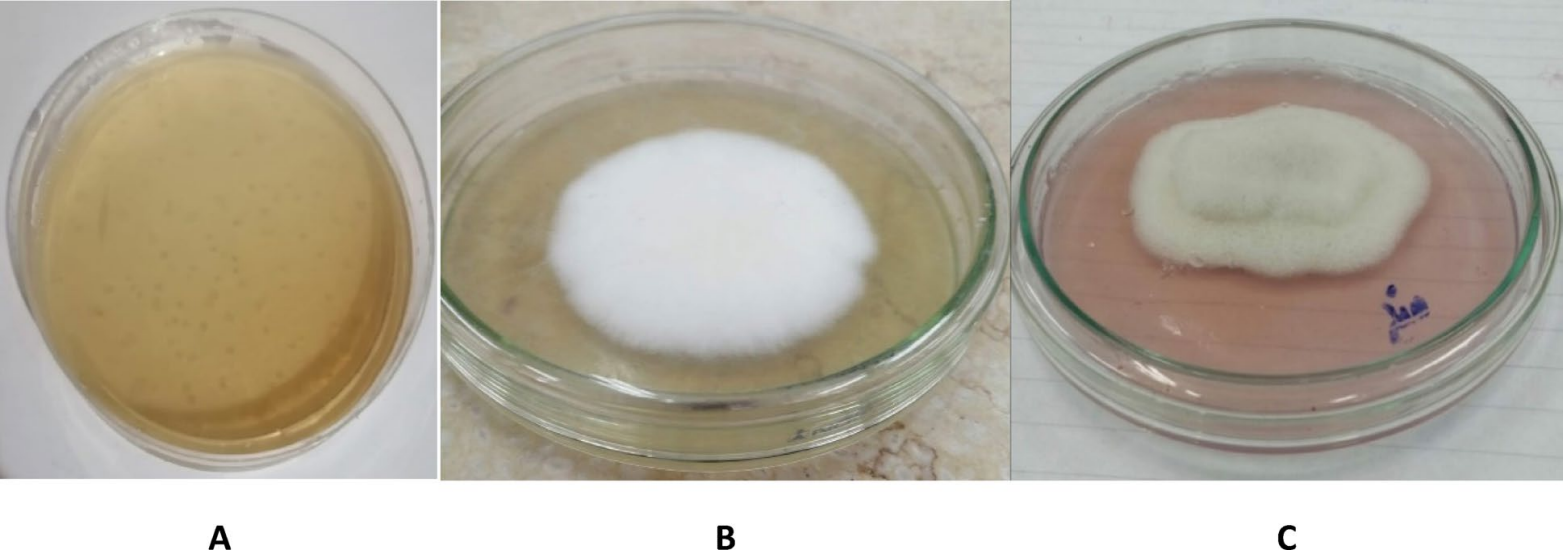
Qualitative L-ASNase activity indicated by pink color on modified Czapek-Dox agar plate. **A**: Negative control, an uninoculated plate using sodium nitrate in the medium, instead of L-asparagine. **B**: Positive control plate inoculated with *p*. *ostreatus*. **C**: *P. ostreatus* showing pink zone of L-ASNase production in the medium

L-ASNase production from *P. ostreatus* AUMC 16015 appeared as a pink zone surrounding the colonies, as shown in Fig. 1. The pink zone diameter around the colony was 4.6 cm, while the colony diameter was 4.4 cm, compared to positive and negative controls, where the medium lacked L-asparagine. Additionally, Sisay et al. [5] reported that sixty-five fungal isolates were obtained based on the formation of halo zones around their colonies growing on MCD culture medium with L-asparagine as the only source of nitrogen. The appearance of the halo zone, marked by a color change from yellow to pink, indicates an increase in the medium’s pH because ammonia molecules are released from L-asparagine hydrolysis catalyzed by the isolates’ L-ASNase [52]. *P. ostreatus* was selected for advanced production study following this demonstrated preliminary activity due to its superior biological and bioprocess benefits. Filamentous fungi like *P. ostreatus* provide strategic advantages compared to Gram-negative bacterial L-ASNase systems. Fungi facilitate extracellular secretion, which simplifies downstream processing and improves biosafety, in contrast to intracellular bacterial enzymes that need lysis and risk lipopolysaccharide (LPS) contamination [53]. Under SSF, the lignocellulolytic machinery of *P. ostreatus* (laccases, manganese peroxidase, cellulases, xylanases) depolymerizes agricultural wastes, allowing controlled carbon release that reduces carbon catabolite repression) CCR [54]. Unlike bacterial systems, *P. ostreatus* is generally recognized as safe (GRAS) eukaryote that provides human-like glycosylation to reduce immunogenicity [55]. This SSF platform demonstrates industrial scalability and mechanistic insight.

### L-ASNase detection in both extracellular or intracellular extracts of *P. ostreatus *

L-ASNase catalytic activity was evaluated in both the cell-free supernatant and in cells disrupted by sonication to quantify enzyme production and determine whether the enzyme was produced intracellularly or extracellularly. The results demonstrated that *P. ostreatus* AUMC 16,015 predominantly secretes L-ASNase extracellularly. Enzyme productivity was calculated depending on the quantity of ammonia that is generated when L-asparagine is hydrolyzed in the fungal filtrate. Quantitative assay revealed that L-ASNase activity in the fungal filtrate reached 18.08 U/mL, whereas a negligible activity was detected in the disrupted cell extracts at 5.1 U/mL. Parallel observations were obtained by [39], who observed that *Fusarium equiseti* predominantly secretes L-ASNase extracellularly, with peak enzyme activity of approximately 12.6 U/mL detected in the culture supernatant. Furthermore, a comprehensive analysis by [2] concluded that L-ASNase is typically secreted extracellularly by species of *Aspergillus*, *Penicillium*, and *Fusarium* during both submerged and solid-state fermentation. In contrast, several studies showed that L-ASNase localized predominantly inside fungal cells [56] demonstrated that when they expressed the *Fusarium proliferatum* L-ASNase gene in *Pichia pastoris* with a secretion signal, most of the enzyme remained intracellular, and cell disruption was required to recover bioactive enzyme, resulting in an eightfold higher activity than the extracellular fraction. Similarly, [8] reported that fungal species, including *Penicillium sizovae* and *Fusarium proliferatum*, from Brazilian savanna soils exhibited measurable L-ASNase activity only after mechanical disruption of mycelial cells, with intracellular enzyme recovery up to five times greater than from culture filtrates.

### Morphological and microscopical identification of*P. ostreatus*

Morphological and microscopical characteristics of *P. ostreatus* appeared as whitish mycelium development on the PDA plate. However, spores are white to lilac gray in mass, cylindrical to oblong in shape, and produced white spores prints when viewed via a compound light microscope.

### Molecular identification of*P. ostreatus*

**Fig. 2:**
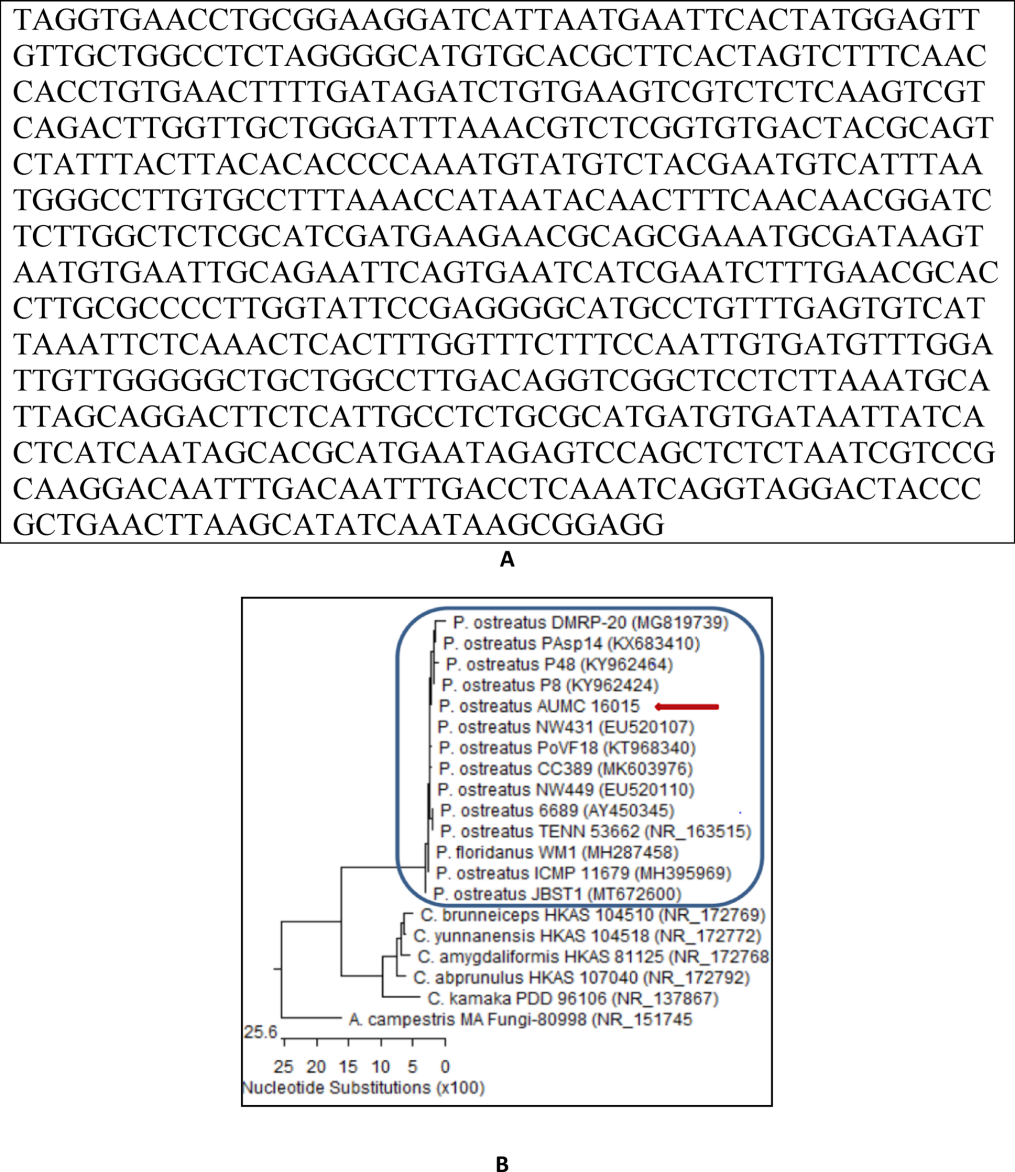
ITS sequences of the rRNA gene of *P. ostreatus* (672 letters) (**A**); phylogenetic tree based on ITS sequences of *P. ostreatus* AUMC 16,015 with accession no. PX089567 (arrowed) aligned with closely related strains accessed from the GenBank. Sequences of *P. ostreatus* AUMC 16,015 showed 99.041% − 100% identity and 92% − 100% coverage with related sequences of the same species, including those of the type material *P. ostreatus* TENN 53,662 (NR163515). Agaricus (*A. campestris*) is included as an outgroup strain. *P* Pleurotus, *C* Clitopilus (**B**)

Molecular identification was conducted to validate the morphological and microscopical confirmation of *P. ostreatus.* Identification with ITS (internal transcribed spacer) patterns (Fig. 2A) and Phylogenetic tree (Fig. 2B). Sequences of *P. ostreatus* AUMC 16,015 (672 letters) showed 99.041% − 100% identity and similarity of about 92% − 100% of the sequences connected to the same species, comprising the material type of *P. ostreatus* AUMC 16,015 TENN 53,662 (NR163515) with Accession No: PX089567. Agaricus (*A. campestris*) is included as an outgroup strain, P. = *Pleurotus*, C. = Clitopilus.

###  Effect of agricultural wastes on L-ASNase production (SSF)

**Fig. 3:**
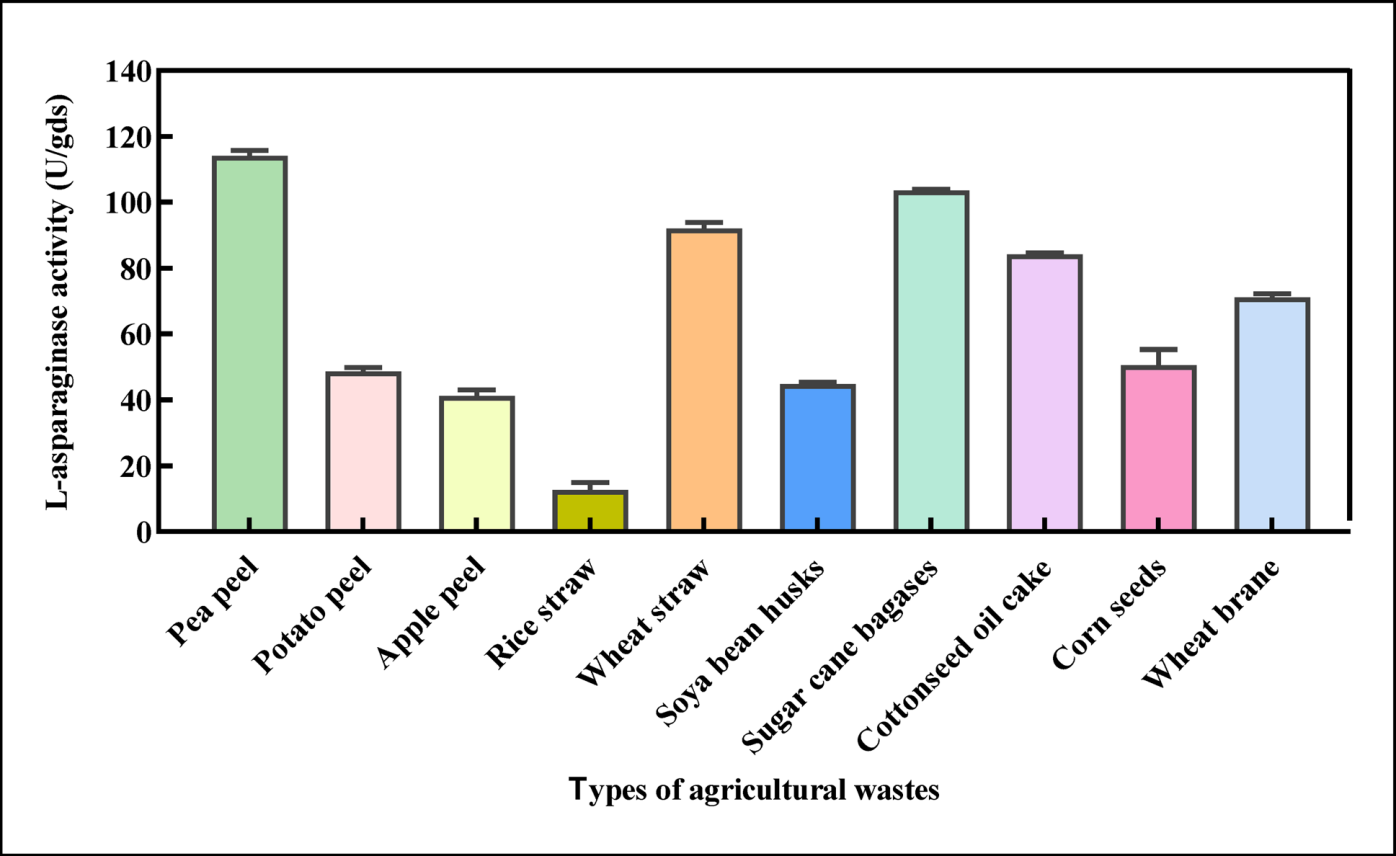
Effect of different agricultural wastes on l-ASNase production of *P. ostreatus* under SSF. The results are illustrated as means ± SD, where the F value indicated a very highly significant difference (F = 20.42, *P* ≤ 0.0001)

Ten different substrates were illustrated in Fig. 3. Out of all substrates, Pea peel was demonstrated to be the most effective substrate for bioproduction of L-ASNase, where the activity was 114.06 U/gds, followed by sugarcane bagasse and wheat straw with production of 103.59 and 92.00 U/gds, respectively. Also, the substrates cottonseed oil cake and wheat bran supported the enzyme production, but at lower levels (84.15 and 71.05 U/gds, respectively). Rice straw yielded the lowest L-ASNase activity, reaching only 12.71 U/gds. The selection of an optimal agricultural waste for SSF primarily depends on its ease of nutrient breakdown and assimilation by the fungus, as well as the substrate’s natural availability and cost-effectiveness [57]. Pea peel outperformed other substrates through its matrix composition and intrinsic bioactivity. Unlike starchy wastes, pea peel (*Pisum sativum L*.) has a rigid lignocellulosic structure (~61% cellulose, 22% lignin) that resists bed compaction, making it an ideal SSF substrate. It facilitates effective fungal colonization without agglomeration due to its high-water absorption index (WAI: 3.24 g/g) [58, 59]. Pea peel is a natural L-ASNase inducer since peas store nitrogen as asparagine, eliminating synthetic supplementation [60]. Polyphenols like 5-caffeoylquinic acid (~59.87 mg/100 g) scavenge ROS and mitigate SSF thermal/oxidative stress without additives [61].

*P. ostreatus* lignocellulolytic enzymes allow controlled depolymerization of recalcitrant substrates, supplying trickle-release sugars to prevent CCR and sustain enzyme production [54]. Pea peel’s inducer-protector dual functionality creates a zero-waste SSF matrix optimized for fungal ASNase production [61]. Another study by [12] demonstrated that Pomegranate peel was the optimal substrate for *Fusarium oxysporum* F-S3 to synthesize L-ASNase, with a maximal enzyme yield of 160.3 U/g dry substrate (gds), followed by potato peel (59.2 U/gds). Pea and banana peel exhibited comparatively lower enzyme yields of 24.3 and 15.4 U/gds, respectively. Several filamentous fungi are potent producers of L-ASNase in SSF using agro-industrial residues. For instance, Meghavarnam and Janakiraman [28] reported that *Fusarium culmorum* cultivated on soybean meal generated a 7.21 U/gds maximal L-ASNase activity. Similarly, [62] found that the optimal substrate for *Aspergillus niger* was passion fruit peel flour, which produced the largest amount of L-ASNase (2380.11 U/gds) in 48 h, or 49.6 U/gds.h.

### Optimization of culture conditions to maximize*P. ostreatus* L-ASNase under SSF

#### Effect of pretreatment of pea peel with water on L-ASNase production by *P. ostreatus *under SSF

The pre-treatment of the selected pea peel with sterilized hot water at 70 °C for 10 min yielded low L-ASNase activity (21.22 U/mL), whereas cold sterilized distilled water resulted in the highest L-ASNase activity, reaching 24.30 U/mL (an unpaired T-test, *P* ≤ 0.05). Consequently, the optimal cold-water method achieved a 1.15-fold increase in enzyme bioproduction compared to the hot-water method. This outcome can be explained by the exposure of agricultural wastes to elevated thermal temperatures, which can lead to nutrient degradation and the formation of inhibitory compounds, which adversely affect fungal mycelial growth and the biosynthesis of essential metabolites. In contrast, conventional treatment of pea peel with distilled water effectively prevents the proliferation of unwanted microorganisms without compromising substrate quality [12]. Another study by [12] compared the efficacy of autoclaving versus gamma irradiation for sterilizing pomegranate peel used as a substrate in SSF. The fungus *F. oxysporum* F-S3 produced L-ASNase under SSF conditions. Upon increasing the gamma irradiation power to 15 kGy, no signs of microbial contamination were identified, and this irradiated substrate yielded significantly more L-ASNase than the autoclaved substrate. This observation aligned with findings previously reported by [12], who showed that the autoclaved substrate produced 1.43 times less L-ASNase compared to the irradiated one. Furthermore, the autoclaved substrate developed a black coloration, whereas the irradiated substrate retained its original color.

#### Effect of the moistened pea peel with the used medium or with water on L-ASNase production by*P. ostreatus*

To achieve the desired moisture type, moistened pea peel with modified Czapek-Dox medium or with sterilized distilled water was evaluated. L-ASNase activity reached a maximum of 25.43 U/mL using 4 mL of freshly prepared modified Czapek-Dox medium, compared to sterilized distilled water (20.56 U/mL) (an unpaired T-test, *P* ≤ 0.05). The modified medium yielded a 1.24-fold increase in enzyme production. Ali et al. [63] reported that the maximum L-ASNase production was achieved in medium supplemented with 1% L-asparagine, 0.2% glucose, 0.1% KH₂PO₄, 0.025% KCl, and 0.052% MgSO₄0.7 H₂O. Under SmF conditions, *A. sydowii* and *F. oxysporum* enzyme activities were measured at 146.0 U/mL and 143 U/mL, respectively. In contrast, [64] observed that water was the most effective moistening agent in SSF across several solid substrates when compared to the mineral-enriched M9 medium. The highest L-ASNase activity for *A. terreus* MTCC 1782 was recorded in water-moistened pomegranate peel (253 U/g dry substrate), following the highest-yielding substrate, water-moistened wheat bran, and M9 medium-moistened coconut oil cake yielded enzyme activities of 110 U/gds and 85 U/gds, respectively.

#### Effect of pea peel particle sizes on L-ASNase production by *P. ostreatus *

**Fig. 4:**
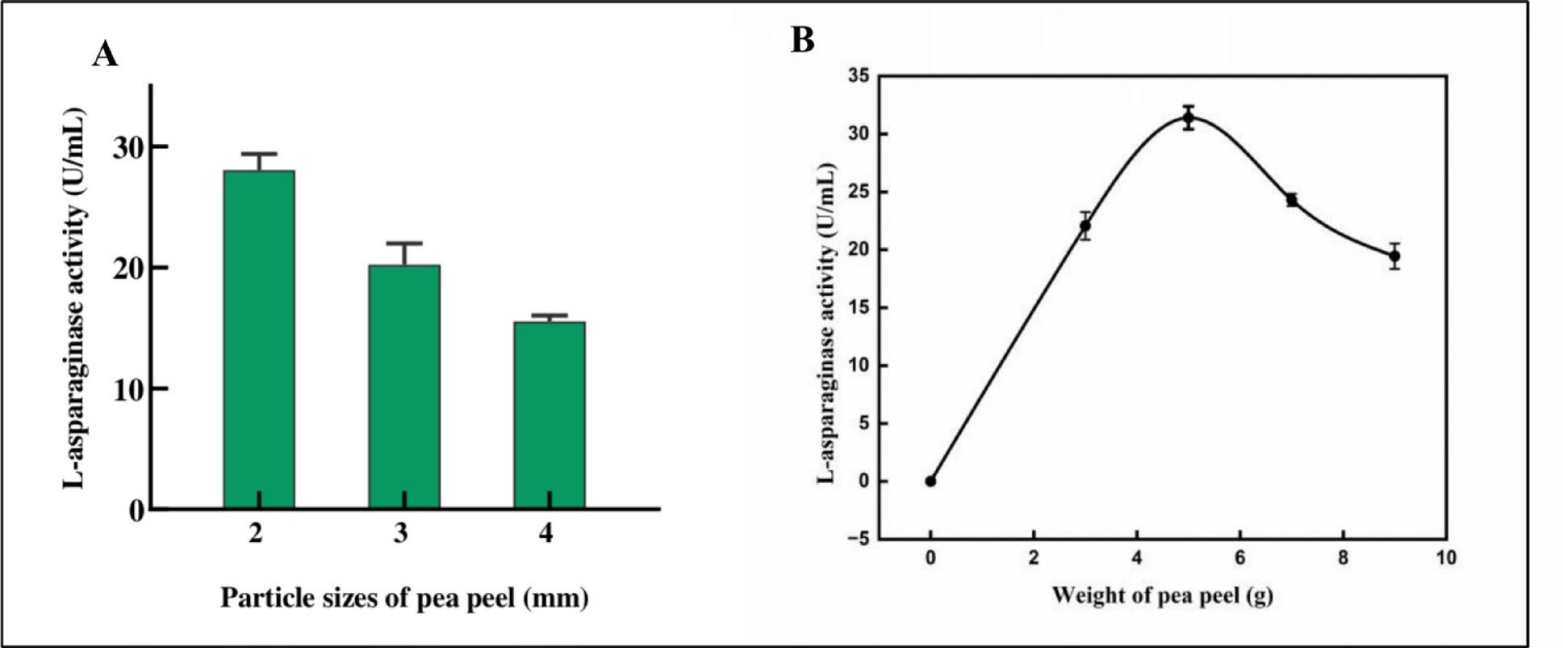
Optimization of culture conditions to maximize *P. ostreatus* L-ASNase under SSF. **A**: Different pea peel particle sizes. **B**: Different pea peel weights. The results are illustrated as means ± SD, where the F value indicated a highly significant difference (*P* ≤ 0.01)

Different particle sizes (2, 3, 4 mm) of the selected pea peel substrate were assessed for their impact on L-ASNase synthesis. The results exhibited that the highest enzyme hydrolytic activity was achieved using fine pea peel particles (2 mm), reaching 28.04 U/mL (Fig. 4A). In comparison, intermediate (3 mm) and large particle sizes (4 mm) yielded the lowest L-ASNase activities of 20.23 U/mL and 15.53 U/mL, respectively. Smaller particle sizes promote microbial enzyme production by increasing surface area and boosting mass transfer, while larger particles limit microbial growth and enzyme biosynthesis due to restricted nutrient and oxygen diffusion [65]. According to Mishra [65], a 1205–1405 μm range for particle size derived from three leguminous crops yielded optimal enzyme production by *Aspergillus niger* during SSF.

#### Effect of pea peel weights on L-ASNase production by*P. ostreatus*

Five pea peel weights (3, 5, 7, 9, and 11 g) were examined to determine the ideal substrate weight for the highest level of L-ASNase assay result. The results indicated that L-ASNase catalytic activity from *P. ostreatus* increased with the weight of pea peel, peaking at a concentration of 5 g, where a maximum activity was 31.41U/mL. L-ASNase activity decreased at substrate weights above and below this optimal concentration (Fig. 4B). This optimal substrate weight ensures adequate nutrient availability and aeration, whereas higher substrate loads lead to decreased enzyme production, likely due to nutrient depletion and restricted microbial growth conditions [66].

#### Effect of moisture contents of pea peel on L-ASNase production by*P. ostreatus*

**Fig. 5:**
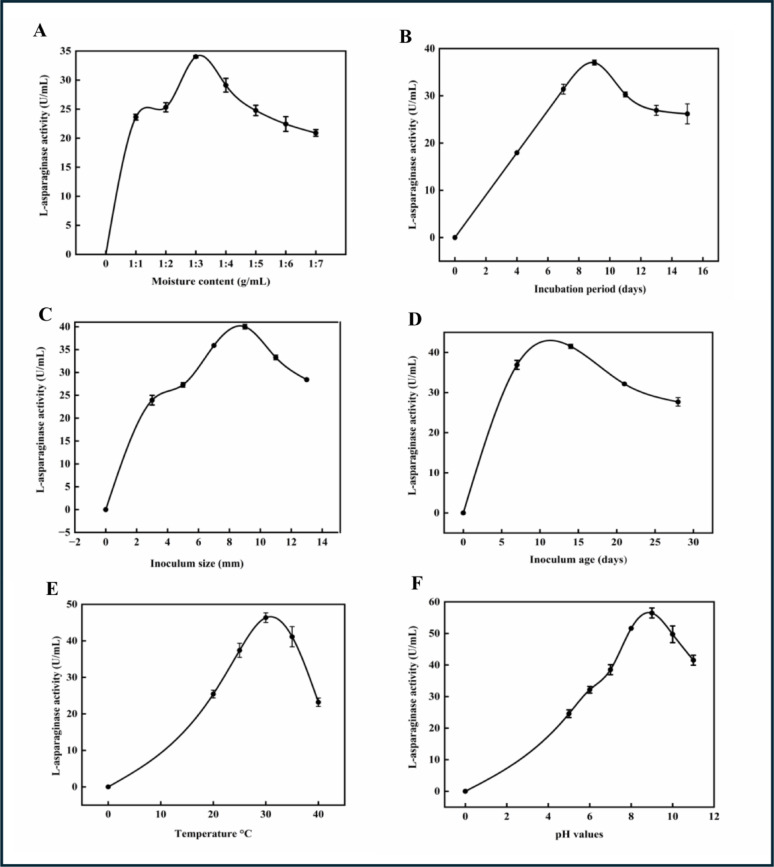
**A**: Different moisture contents. **B**: Different incubation periods. **C**: Different inoculum sizes. **D**: Different inoculum ages. **E**: Different incubation temperatures. **F**: Different pH values. The results are illustrated as means ± SD, where the F value indicated a very highly significant difference (*P* ≤ 0.001)

This test was evaluated using the initial moisture content of pea peel at different ratios (1:1–1:7) (g/mL). The maximum activity of *P. ostreatus* L-ASNase was 34.03 U/mL at a 1:3 (g/mL) moisture ratio, representing 75% moisture (Fig. 5A). It became obvious that the enzyme activity declined as this ratio increased or decreased. The production and synthesis of new cells depend on a specific amount of water. When water content is reduced, L-ASNase production may decrease due to reductions in nutrient dissolution, substrate swelling, and water absorption for the solid substrate. Conversely, increasing the moisture content excessively can impair oxygen transfer, leading to a decline in gas volume and a reduction in gaseous exchange. It also causes changes in lignin decomposition, alterations in the structure and porosity of substrate particles, increases the risk of bacterial contamination, and reduces fungal growth [67]. In parallel to our study, Elshafei and El-Ghonmey [68] discovered that *Trichoderma viride* F2 produced the most L-ASNase production at 75% moisture content (84.68 U/gds). Meghavarnam and Janakiraman [28] reported that *F. culmorum* (ASP-87) yielded a 7.33 U/gds maximum L-ASNase activity at a 70% moisture content using agricultural raw materials. Conversely, Isac and Abu-Tahon [69] achieved the maximum L-ASNase *F. solani* AUMC 8615 output at 60% initial moisture content of 400.5 U/mL. A moisture content of 85.7% was achieved by adjusting the wheat bran: water ratio to 1:6, L-ASNase *Rhizopus oryzae* AM16 secretion levels steadily to 2,509.6 U by Othman et al. [67].

#### Effect of incubation periods on L-ASNase production by*P. ostreatus*

The inoculated flasks were incubated for several incubation periods (4, 7, 9, 11, 13, and 15 days). The quantity produced of L-ASNase peaked after 9 days of incubation, reaching 37.02 U/mL. After the 9th day of incubation, enzyme activity gradually declined (Fig. 5B), as the culture likely entered a stationary phase. This decrease in activity could be due to a depletion of nutrients, which can no longer support constant growth, or it may be attributed to proteolytic activity, causing the enzyme to become inactive [70]. Enzyme activity decreases with longer incubation times and could be carried through nutrient depletion or the accumulation of hazardous byproducts [71]. Patro and Gupta [42] reported similar results on *Penicillium* sp., where ten days were the ideal incubation period for maximum L-ASNase production. Moreover, Chinnadurai and Govindasamy [72] showed eight days of optimal incubation for *Aspergillus* species. According to Bedaiwy [32] *P. ostreatus* exhibited L-asparagine amidohydrolase activity, which peaked at 6.4 U/mL after 7 days of incubation. Under SSF. On the contrary, another previous study by Doriya and Kumar [73] investigated the production of *Aspergillus* sp. L-ASNase increased gradually, peaking on day six; following a substrate mix of 500 g and 1000 g, respectively, the enzyme activity was 5.41 and 6.67 U/mL, under SSF. Furthermore, a study on *Aspergillus fumigatus* by Dutta et al. [57] observed that the optimal incubation duration was four days, yielding 102.9 U/mL under SSF. Eisele et al. [74] investigated that the optimum incubation time was 18 days for *Flammulina velutipes* L-ASNase under SmF.

#### Effect of inoculum sizes on L-ASNase production by*P. ostreatus*

The culture medium was inoculated with (3, 5, 7, 9, 11, and 13 mycelial plugs (5 mm)) of *P. ostreatus* under aseptic conditions, with a rising inoculum size, L-ASNase activity increased, reaching maximum productivity of 40.01U/mL at nine mycelial plugs (Fig. 5C). Below and above this size, L-ASNase activity declined. While very low inoculum levels result in insufficient biomass, leading to poor growth of the producer strain and low enzyme yield, increasing the inoculum size improves enzyme synthesis. In contrast, a significantly larger inoculum amount might exhaust the nutrients in the substrate and generate excessive biomass, resulting in competitive inhibition of nutrients [75]. Pallem et al. [76] reported that 4.81 U/mL was the ideal L-ASNase yield in a previous study using a 1.5 mL inoculum volume of *F. oxysporum* at 7 days of growth. Although Zia et al. [77] discovered that the greatest activity of L-ASNase was obtained with a 5% inoculum volume of *A. niger*, reaching 5.40 U/mL, whereas an increase in inoculum size resulted in spore overcrowding, which reduced the enzyme activity. In addition, da Cunha et al. [62] reported that the optimal conditions yielded the maximum L-ASNase assay result of passion fruit peel flour with an inoculum density of 2.1 × 10^6^ spores/g. A study by Parashiva et al. [78] identified the optimum inoculum concentration as 1.5 × 10^6^ spores/mL for maximum *Fusarium foetens* L-ASNase activity.

#### Effect of inoculum ages on L-ASNase production by*P. ostreatus* under SSF

The ideal conditions were changed after inoculating this culture medium with varied ages (7, 14, 21, and 28 days). According to the data, L-ASNase’s maximal activity was 41.51 U/mL on the 14th day for the inoculum age of *P. ostreatus* (Fig. 5D). A previous study by Bedaiwy [32] proved that the inoculum’s age had the greatest impact on *P. ostreatus* L-ASNase catalytic activity, recorded at 7.89 U/mL on the ninth day.

#### Effect of temperatures on L-ASNase production of*P. ostreatus*

The incubation temperature is one of the most crucial factors in producing L-ASNase because it affects the rate of enzymatic activity by influencing microbial growth, enzyme release, and the rate of the chemical reaction [79]. The present study investigated L-ASNase production across five incubation temperatures: 20, 25, 30, 35, and 40 °C. The ideal L-ASNase activity markedly increased from 25.43 U/mL at 20 °C to 46.37 U/mL at 30 °C (Fig. 5E). Further temperature increases resulted in a decrease in productivity until it reached 23.18 U/mL at 40 °C. Significantly less enzyme activity was produced by further raising the temperature. This could be because of the medium’s buildup of heat, followed by the enzyme’s denaturation [80]. Similar findings were reported by Sharma and Mishra [81], who observed maximum enzyme productivity of L-ASNase from *Aspergillus niger* at 30 °C. Another study by Isaac and Abu-Tahon [69] reported that the optimal temperature for L-ASNase activity from *Fusarium* sp. at 30 °C generated a maximal activity equal to 190.0 U/mL under SSF. Conversely, Ahmed et al. [13] found that the optimal incubation temperature of 27 °C produced the highest yields of *Aspergillus* sp. L-ASNase under SSF. Doriya and Kumar [73] investigated that contrast, Sisay et al. [5] also investigated that *Candida palmioleophila* L-ASNase demonstrated the highest production (43.8 U/mL) at incubation temp. 25 °C, while *Trichosporon asahii* L-ASNase demonstrated 54.7 U/mL at 35 °C, respectively. L-ASNase derived from the majority of microbes exhibited a maximal temperature. range between 30 and 40 °C; On the other hand, Zuo et al. [82] found that Thermostable L-ASNase was synthesized by *Thermococcus. kodakaraensis* TK1656 and *T. gammatolerans* EJ3 at 85 °C.

#### **E**ffect of pH on L-ASNase production by *P. ostreatus*

Since raising the culturing medium’s pH is essential to raising L-ASNase production, the impacts of five starting pH values (5, 6, 7, 8, and 9) were examined. According to the present results, the optimum pH for maximum L-ASNase productivity from *P. ostreatus* was 56.47 U/mL at pH 9. Study results showed that modifying pH levels from 9 to acidity or alkalinity significantly reduced production (Fig. 5F). One important factor influencing the generation of enzymes is the initial pH of the enzyme-producing medium. By controlling the amount of nutrients available in the medium, it can have an indirect effect on fungal development. After the pH reaches its ideal level, a decrease in enzyme activity may result from the enzyme’s partial inactivation, which occurs when the ionizable groups of the enzyme separate. So, a change in pH stops a substrate from attaching to the enzyme because it alters the structure and characteristics of the enzyme and the substrate [83]. In parallel to our study, Rahiman et al. [84] showed that pH 9.0 was the ideal pH for *A. nige*r*’s* L-asparagine amidohydrolase activity. Isaac and Abu-Tahon [69] investigated the highest amount of L-ASNase from *F. solani* AUMC 8615 produced (302 U/mL) at an initial pH of 8.0. Also, this result was consistent with studies by Vijay and Raju [31], who found that the maximum L-ASNase *A. terreus* MTCC 1782 production of 191.3 (U/gds) was obtained at pH 8.0 under SSF conditions. In contrast, Fernandes et al. [85] found that the greatest yield of the enzyme for *Penicillium solitum* was found at pH 7, while for *Aspergillus caespitous*, the best yield was at pH 6.0. In another study, Bedaiwy [32] found that *P. ostreatus* produced L-ASNase with high enzymatic activity (7.27 U/mg) at an initial pH of 6.0.

### Purification of *P. ostreatus* L-ASNase

**Fig. 6:**
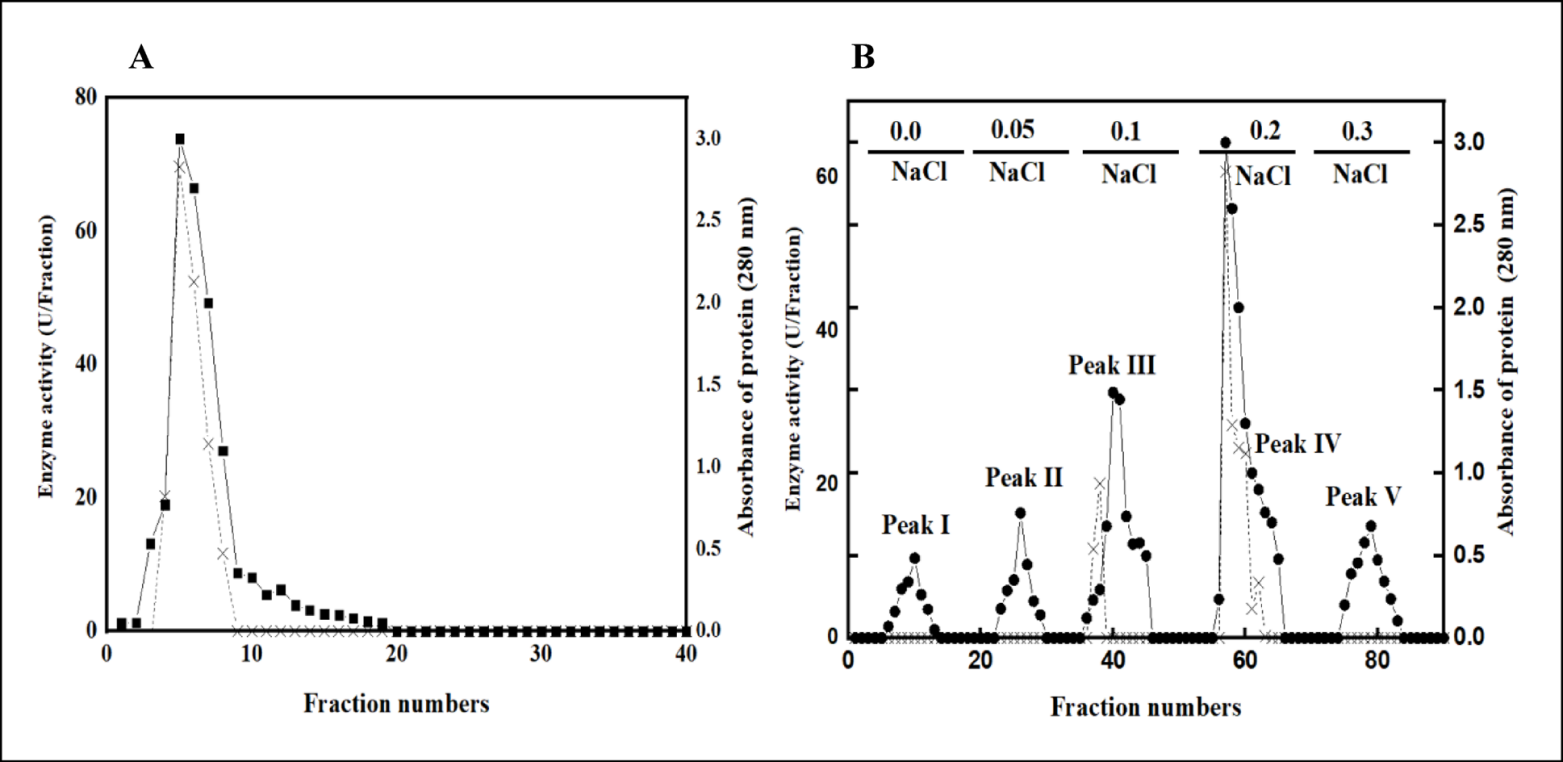
**A**: *P. ostreatus* L-ASNase elution profile using Sephadex G-150 gel-filtration column. **B**: *P. ostreatus* L-ASNase elution profile using DEAE cellulose ion exchange column

The results of *P. ostreatus* L-ASNase purification were summarized as shown in Table 1. The first purification step by ammonium sulfate precipitation (80%) achieved a 2.95-fold purification, yielding a specific enzyme activity of 1012.64 U/mg and a percentage yield of 73.33%. For further purification of L-ASNase, (Fig. 6A) showed Sephadex G-150 size exclusion chromatography column was carried out. The enzyme was purified about 3.30 times in specific activity of Sephadex G-150 column, rising from 1012.64 to 1130.27 U/mg with a yield rate of up to 41.66%. The final purification step was achieved via ion exchange chromatography employing DEAE cellulose, as illustrated by Fig. 6B. This purification stage yielded a 5.22-fold enhancement in enzyme activity, achieving an overall activity of 1801.97 U/mg and a recovery rate of 23.25%. The findings of the present investigation were comparable to previously published research by [86] for *A. niger* and *A. quadrilineatus* L-ASNase purification. Enzymes from *A. niger* displayed 2.38-fold, 14.06% yield, and *A. quadrilineatus* enzyme with 2.39-fold, 13.92% yield. Conversely, [41] documented that 65% (NH_4_)_2_SO_4_ precipitation, Sephadex G-100 gel filtration, and ion-exchange chromatography were used to purify marine *A. terreus* L-ASNase, yielding an 11.96-times purification and 14.22% yield. Moreover, [87] observed that L-asparagine amidohydrolase of *A. oryzae* CCT 3940 was purified to a 28.6-fold increase with a 6.0% yield following sulfate fractionation and chromatographic purification using Q Sepharose^™^, SP Sepharose^™^, and CM Sepharose^™^ columns. Additionally, L-ASNase from endophytic fungus *Lasiodiplodia theobromae* was purified with a 13.68-fold, 26.79% yield by [40]. According to a study by [88], an approximate 55% yield and 70.9-fold purification were achieved after four purification steps of extracellularly produced L-ASNase derived from *Erwinia chrysanthemi*.

**Table 1:** Purification profile of L-ASNase from* P. ostreatus *

Purification step	Parameter				
	Total activity (U)	Total protein (mg)	Specific activity (U/mg)	Fold	Yield (%)
Crude isolated enzyme	897.57	2.62	345.22	1.00	100
Ammonium sulphate (80%)	658.21	0.65	1012.64	2.96	73.33
Sephadex G-150	373.99	0.33	1130.27	3.30	41.66
DEAE cellulose	208.68	0.12	1801.97	5.22	23.25

### Molecular weight estimation

**Fig. 7:**
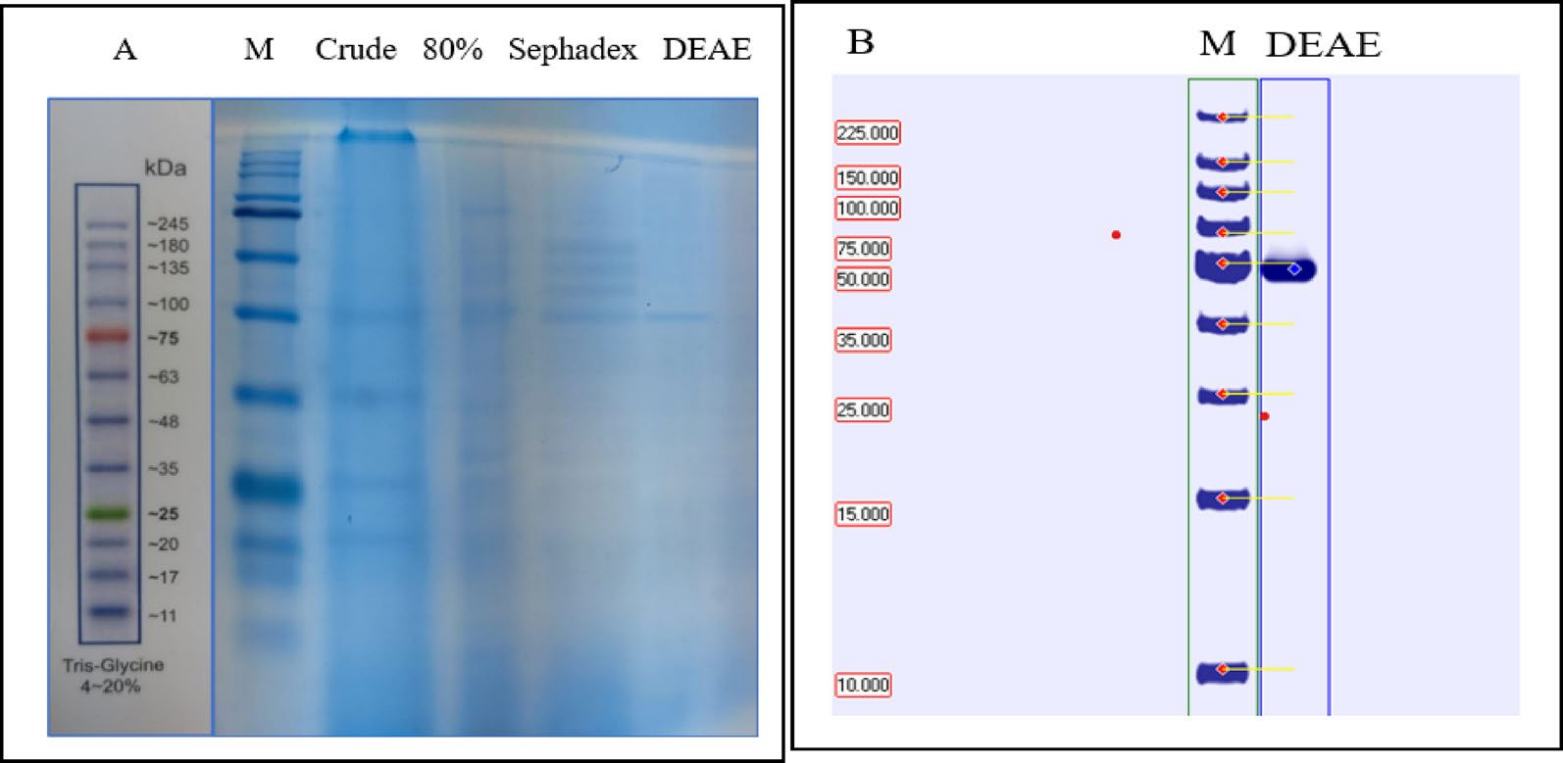
**A**: SDS-PAGE electrophoresis. The molecular weight of standards (Lane 1; 245, 180, 135, 100, 75, 63, 48, 35, 25, 20, 17, 11kDa), the crude extract (Lane 2), ammonium sulfate 80% precipitated protein (Lane 3), L-ASNase fractions were obtained from the Sephadex G-150 column (Lane 4) and purified L-ASNase fraction obtained from DEAE cellulose column (Lane 5). **B**: Native polyacrylamide gel electrophoresis, the molecular weight of standards (Lane 1; 225, 150, 100, 75, 50, 5, 25, 15, 10 kDa) and the purified L-ASNase that was obtained from the DEAE cellulose column

SDS–PAGE analysis of pure L-asparagine amidohydrolase at various purification stages demonstrated progressively clearer electrophoretic bands from the crude extract to the final ion-exchange DEAE cellulose chromatography step. It exhibited a single distinct protein band corresponding to L-ASNase in pure form with an estimated molecular weight of approximately 48 kDa, as shown in Fig. 7A. Native PAGE has exhibited an approximate molecular weight of 48 kDa (Fig. 7B), which illustrated that the purified L-ASNase of *P. ostreatus* was homogeneous. A similar observation by [39] showed the molecular mass of the purified *F. equiseti* AHMF4 enzyme, as determined by SDS, was approximately 45.7 kDa. Also, [89] showed that the L-ASNase molecular mass generated *by A. niger* was 48 kDa. Also, *A. niger* generated L-ASNase, which had a molecular weight of 48 kDa by [42]. Conversely, Yassein and El-Shahir [86] reported 27.8 kDa and 50.4 kDa for *A. quadrilineatus* and *A. niger*, respectively. L-ASNase had a molecular weight of roughly 96.32 kDa from *Mucor hiemalis* reported by [83]. L-ASNase was thus identified as a homogeneous single-chain protein. However, regarding the tetrameric nature of L-ASNase, the isolated enzyme most likely occurs as a homotetrameric protein complex, given that only a single band was observed [90]. Additionally, the mesophilic fungus’s L-ASNase was discovered to have a greater molecular weight of 216 kDa *Cylindrocarpon obtusisporum* by [91]. Eisele et al. [74] determined that the molecular weight was 85 kDa with 13 kDa subunits, indicating that the enzyme from *F. velutipes* is a hexameric protein [92] purified L-ASNase homodimer from *Rhizomucor miehei* exhibited a native molecular mass of 133.5 kDa and migrated as a single homogeneous band of 72 kDa on SDS-polyacrylamide gel, consistent with its predicted molecular mass of 75,316 Da. While [93] identified the L-ASNase dimeric, which was isolated from *Erwinia carotovora* MTCC 1428 and has two different bands with molecular weights of 40.2 and 39.8 kDa (as heterodimeric).

### Characterization of pure *P. ostreatus* L-ASNase

####  Effect of pH values on the purified L-ASNase activity and stability

**Fig. 8:**
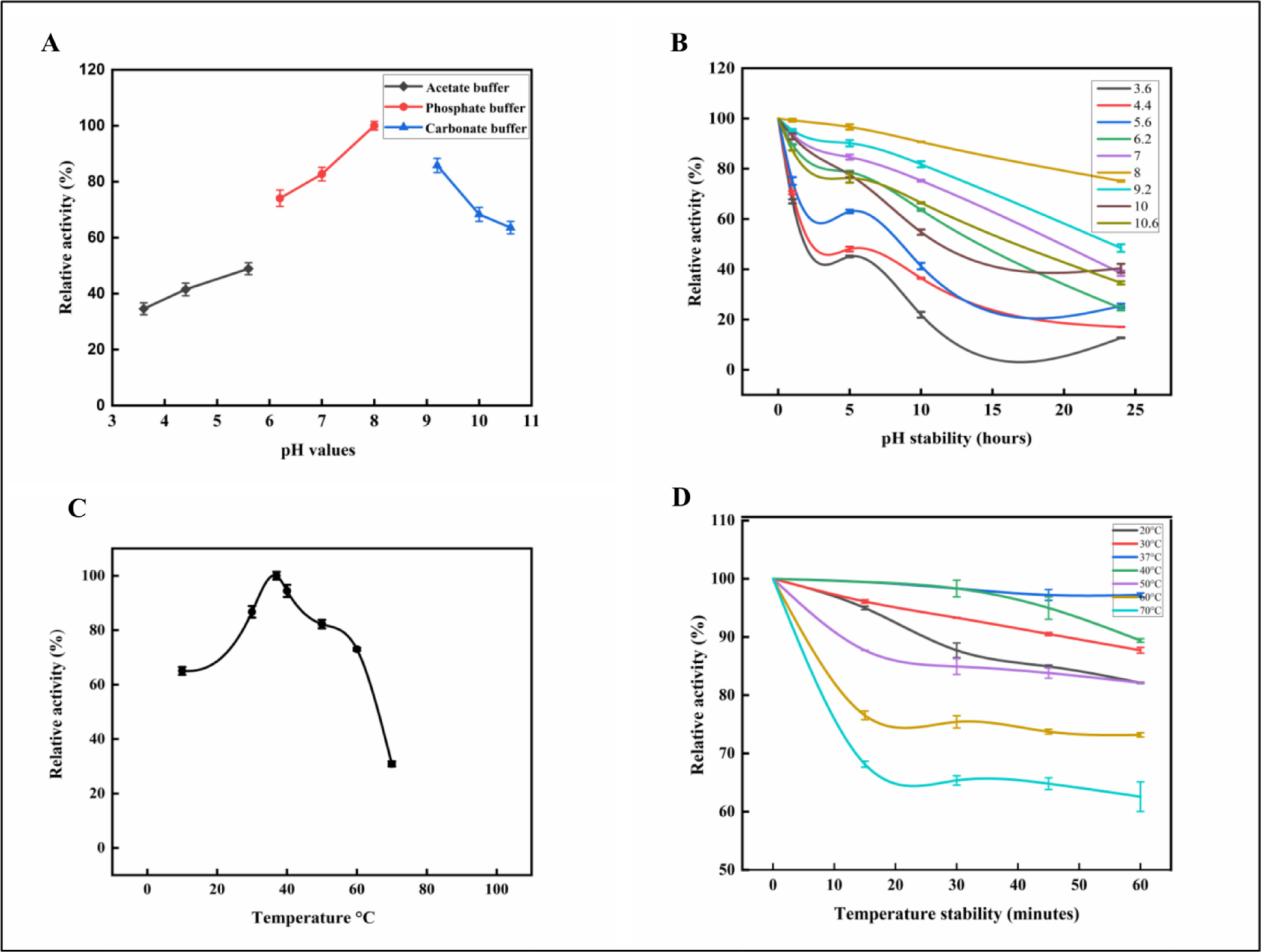
Effect of different pH values and temperatures on the purified L-ASNase relative activity and stability. **A**: Different pH values. **B**: pH stability. **C**: Different temperatures. **D**: Temperature stability. The results were illustrated as means ± SD, where the F value indicated a very highly significant difference (*P* ≤ 0.001)

The activity and stability of purified enzymes were affected by pH because it affects the ionic form of the active site [41]. Pure L-ASNase was more active at an alkaline pH than at an acidic pH. Above and below the optimum pH showed a notable decline in enzyme activity (Fig. 8A). At pH 8, the L-ASNase activity retained 100% of its relative activity; starting from pH 6.2, the enzyme retained 74.07%. In contrast, at pH 7, 9.2 retained 82.71%, and 85.80% of relative activity, respectively. Most L-ASNases isolated from microbial sources operate optimally in an alkali medium, with a reported pH range of 8 to 10 [3]. The enzyme’s optimal alkaline pH is due to the low affinity of the liberated aspartate for the active catalytic site, which allows for continued asparagine binding and hydrolysis. Conversely, at an acidic pH, the breakdown of asparagine produces aspartic acid, which binds firmly and actively to the catalytic region. This strong binding inhibits further asparagine from attaching, thereby reducing enzyme activity [94]. These findings corroborated the present outcomes, in which the highest levels of L-ASNase activity were observed at pH 8. The identical results were also documented for *A. fumigatus*.by [95]. Also [69], Kumar Meghavarnam and Janakiraman [38, 96], and [97] reported that the best L-ASNase activity was achieved at alkaline pH (pH 8) from *F. solani* AUMC 8615, *F. culmorum* ASP-87, *T. viride*, and *A. terreus*, respectively. While Vala et al. [40, 98] reported that *A. niger* AKV-MKBUA and *L. theobromae* had the maximum L-ASNase activity at pH 7.0 and pH 6.0, respectively. Endophytic *Aspergillus* sp. showed two forms of this enzyme, and the best L-ASNase hydrolytic efficiency was achieved at pH 6.0 and 10.0 by [13]. The results demonstrated that the pH activity and pH stability curves closely corresponded and reported that pure L-ASNase demonstrated the greatest stability in a phosphate buffer at pH 8. Additionally, in the present study, L-ASNase retained 84.62% and 38.43% of its initial activity at pH 7.0 after 5 and 24 h of incubation at 37 °C in phosphate buffer, respectively. Greater stability occurred at alkaline pH values, with L-ASNase retaining 96.64% and 75.14% relative activity at pH 8.0, and 9.0.

7% and 48.43% at pH 9.2 after 5 and 24 h, respectively, as in Fig. 8B. Similarly, Isaac and Abu-Tahon [69] proved that the optimal pH for L-ASNase yield produced by *F. solani* AUMC 8615 was determined to be 8.0. Despite this, the enzyme’s stability was reduced to 35% following a 2 h incubation at 37 °C. Dange and Peshwe [99] showed that at pH 9.0, *A. aculeatus* L-ASNase remained stable for 8 h.

####  Effect of temperatures on the purified L-ASNase activity and stability

The effect of temperature on the activity of pure L-ASNase was evaluated across a temperature range from 20 to 70 °C. The data showed that pure L-ASNase showed activity over a wide range of temperatures from 20 to 60 °C. As the temperature rose, L-asparagine amidohydrolase activity increased, reaching its maximum activity at 37 °C with 100% relative activity, followed by 40 °C with 94.4% as shown in Fig. 8C. While at 70 °C, it was significantly reduced, losing approximately 69.2% of its overall relative activity. The findings were consistent with earlier studies by [38, 40, 100], found that the L-ASNase activity of *L. theobromae*, *P. crustosum* NMKA 511, and *M. hiemalis* was highest at 37 °C, 36.9 °C, and 37 °C, respectively. In contrast, [41, 101], and [102] found that 40 °C was the ideal temperature for maximal catalytic efficiency of L-ASNase found in *F. solani* endophyte, *A. terreus*, and *A. nidulans*. while for *A. fumigates* W L002 and *(A) oryzae* CCT 3940, the optimum L-ASNase temperature was at 50 °C by [57] and [87]. The results were also aligned with those of [103] and [45], who concluded that 37 °C was the ideal temperature for the maximum L-ASNase activity from *Bacillus licheniformis* and *(B) licheniformis* ASN51. Also [104] and [105] found that L-ASNase exhibited maximum catalytic activity at 40 °C from *Spirulina maxima* and *Acinetobacter* soli. Additionally, the present study demonstrated that the pure L-ASNase ‘s heat stability from *P. ostreatus* was 97.2% at 37 °C after 60 min. At 40 °C and 50 °C, it retained 89.3 and 81.5% of its relative activity. On the other hand, at 60 and 70 °C, it retained 73.1% and 62.60%, respectively (Fig. 8D). Similarly, the present results were close to L-asparagine amidohydrolase created by marine *A. terreus* by [41]. After 60 min, L-ASNase showed high thermal stability, retaining 89.54% of its activity at 50°C. It maintained 81.60% and 62.47% of its activity at 60 °C and 70 °C, respectively. Furthermore, L-ASNase from *Penicillium brevicompactum* NRC 829 demonstrated complete stability, maintaining 100% activity for 1 h at 37 °C [68]. On the contrary, [13] reported the maximum thermal stability of *Aspergillus* sp. L-ASNases at (30, 40, and 50 °C) by (100, 100, and 111%), respectively. L-ASNase activities dropped by 30% following a 60-min incubation period at 90°C. Moreover, [99] demonstrated that *A. aculeatus* purified L-ASNase remained stable for 2 h at 30°C.

####  Effect of different compounds and surfactants on L-ASNase activity

**Fig. 9:**
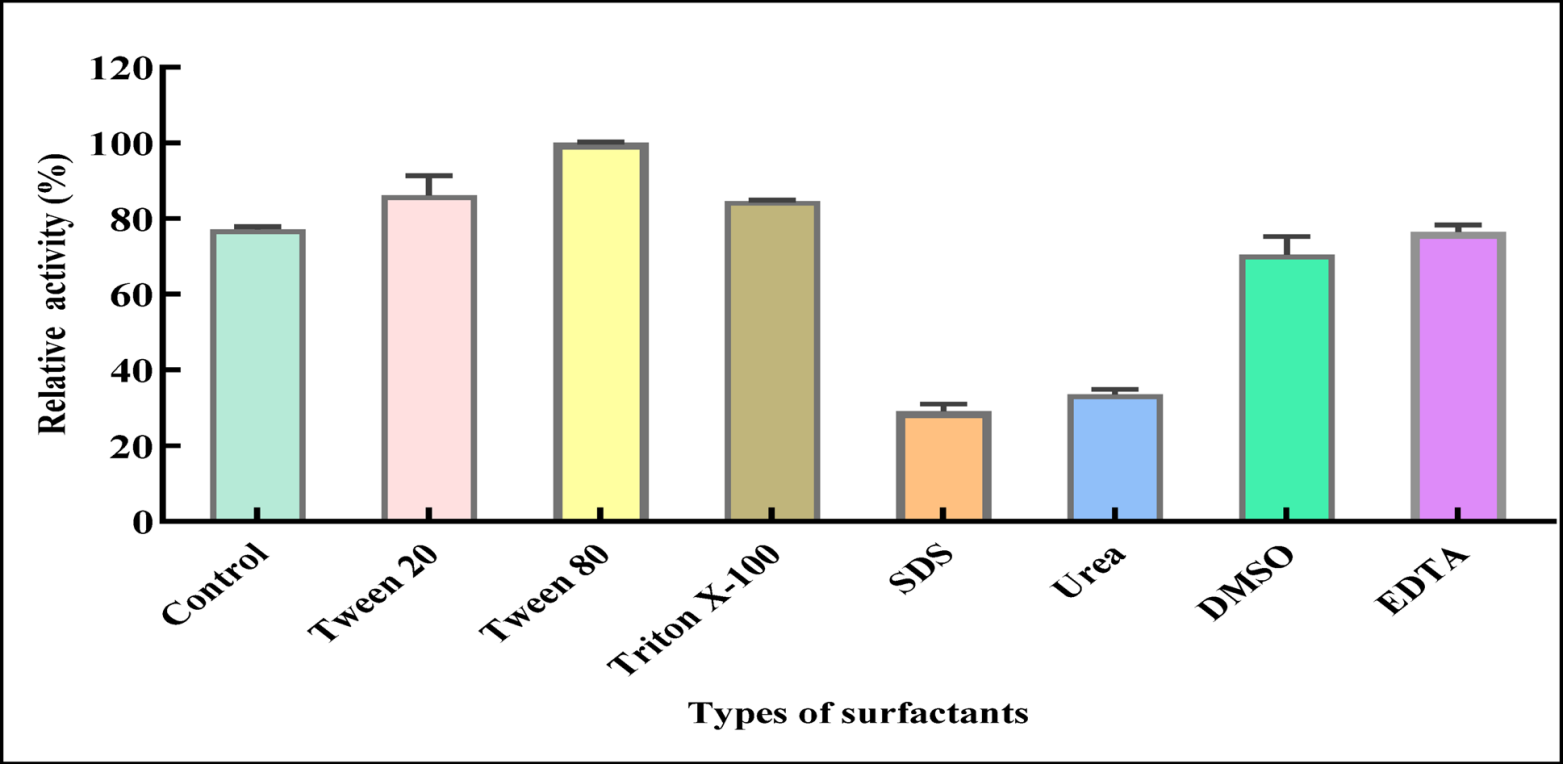
Effect of different types of surfactants on the purified L-ASNase relative activity. The results were illustrated as means ± SD, where the F value indicated a very highly significant difference (F = 170.7, *P* ≤ 0.0001)

The effects of some compounds and surfactants on enzyme activity revealed that L-ASNase exhibited considerable stability to some compounds. SDS recorded a remarkable reduction in L-ASNase activity to 29.11% relative activity while adding urea as a reducing agent; L-ASNase bioactivity was inhibited to 33.61% of its relative activity. L-ASNase showed moderate stability with DMSO at 70.41%. Notably, the surfactants Tween 80, Tween 20, and Triton X-100 enhanced the relative activity to 100%, 86.12%, and 84.61%, respectively, compared to the control without any compounds or surfactant added, which exhibited 77.16% (Fig. 9). Previous studies by [40] agreed with our result, reporting that SDS was the sole reagent that kept 0.65% of the relative activity while demonstrating a decrease in enzyme activity. It does this by breaking up the protein structure, causing a linear model of the folded protein and uniformly negative charges covering the protein, concealing the charges inside it. Relative activity increased by 170% and 180% for Tween 80 and Triton X-100, respectively. Also, the present results were similar to those of [83] for *M. hiemalis* L-ASNase by using (Triton X-100, Tween-80, and SDS), while urea did not have any effect. The present results indicated that the enzyme’s activity remained unaffected by the presence of EDTA, indicating that it is a non-metalloenzyme. L-ASNase can be classified as a metal-activated enzyme rather than a metalloenzyme, as in [83]. Also, the present results were in parallel with those of [13] and [106] for *Aspergillus* sp. and *Cladosporium* sp., respectively. Ahmed et al. [13] also reported the effect of different concentrations of EDTA on the activity of partially pure L-ASNase, which revealed that EDTA did not affect L-ASNase activities from *Aspergillus* sp. This means that enzyme activity was independent of divalent or trivalent ions. On the contrary, [38] found that EDTA inhibited L-ASNase activity by 88% from *T. vidide.*

####  Effect of metal ions on purified L-ASNase activity

**Fig. 10:**
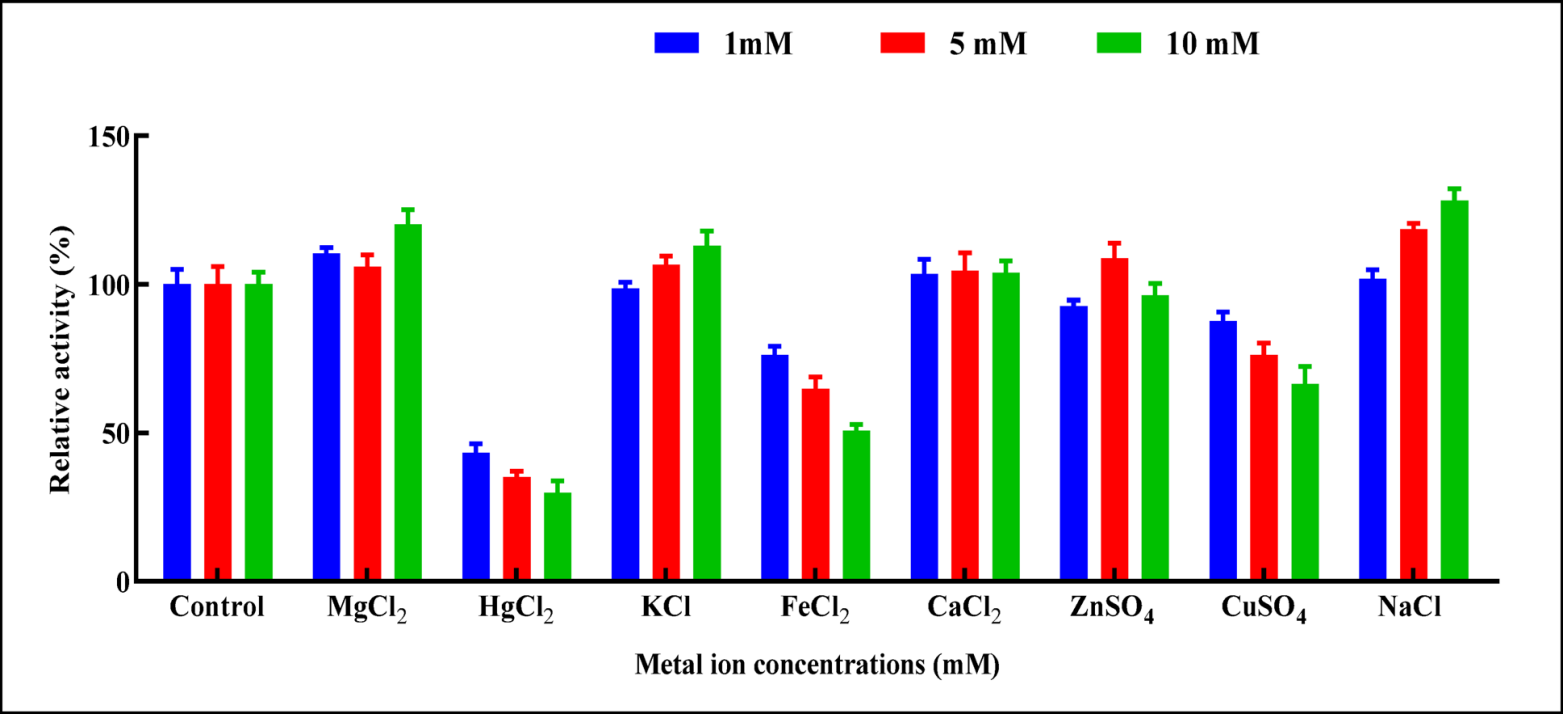
Effect of different metal ion concentrations on the purified L-ASNase relative activity. The results were illustrated as means ± SD, where the F-values indicated very highly significant differences for MgCl₂ (F = 192.7, *P* ≤ 0.001), FeCl₂ (F = 194.2, *P* ≤ 0.001), and NaCl (F = 168.9, *P* ≤ 0.001); highly significant differences for HgCl₂ (F = 52.35, *P* ≤ 0.01), KCl (F = 59.21, *P* ≤ 0.01), and CuSO₄ (F = 47.16, *P* ≤ 0.01); a significant difference for ZnSO₄ (F = 24.69, *P* ≤ 0.05); and no significant difference for CaCl₂ (F = 0.2203, *P* > 0.05)

Among the tested metals shown in Fig. 10, Na^+^ at concentrations of 5 and 10 mM exhibited the highest relative activity of L-ASNase compared to the control without metals (118.60% and 128.19%, respectively). Following, Mg^+ 2^ at 10 mM and K^+^ at 5 and 10 mM showed 120.24%, 106.55%, and 112.9%, respectively. In a parallel study, [41] showed that a 10.0 mM concentration of Mg^+ 2^ and K^+^ elevated L-ASNase enzymatic function by 27.33% and 37.16%, respectively. Also, Na^+^ highly optimized *A. terreus* L-ASNase assay result. Also, [87] revealed that the pure L-ASNase generated by *A. oryzae* was stimulated in the presence of Mg^+ 2^ and Mn^+ 2^ at a 5 mM level. Mg²⁺ ions may activate L-ASNase via improving substrate binding or activation at the catalytic site of the enzyme, where the substrate is directly attached to the enzyme-substrate complex, resulting in the quick release of reaction products [107]. The present results showed that the presence of Na^+^, K^+^, Zn^+ 2^ at 1, 5, and 5mM exhibited a slight positive effect on L-ASNase relative activity (101.96, 106.55, 108.77%), respectively. Also, Ca^+ 2^ at 1, 5, and 10 mM exhibited a weak positive effect (103.44, 104.59, 103.93%), respectively. A slight decline in L-ASNase relative activity was documented in the case of K^+^ at 1mM, Zn^+ 2^ at 1 and 10 mM to 98.7, 92.69, 96.3%, respectively. The highest reductions in relative yield were observed in the presence of Hg²⁺, Fe²⁺, and Cu²⁺ ions at concentrations of 1, 5, and 10 mM, respectively. Specifically, Hg²⁺ and Fe²⁺ recorded reductions to 43.2%, 35.1%, 29.7% and 76.2%, 64.7%, 50.81% respectively; while Cu²⁺ resulted in reductions to 87.7%, 76.22%, and 66.39%. Moreover, in a parallel study [108] showed that Hg^+ 2^ demonstrated a partial suppression of *Vigna unguiculata*’s asparaginase production. As elucidated by [106] the presence of Hg^+ 2^, Cu^+ 2^, Mg^+ 2^, and Zn^+ 2^ reduced the activity of the enzyme, which indicated that the enzyme needs these vicinal sulfhydryl groups for effective catalysis. A comparable observation was similarly made by [109]. Conversely, Cu^+ 2^ improved the fusing strain AYA 20 − 1’s enzymatic activity to 106% as described by [110].

#### Effect of substrate specificity

**Fig. 11:**
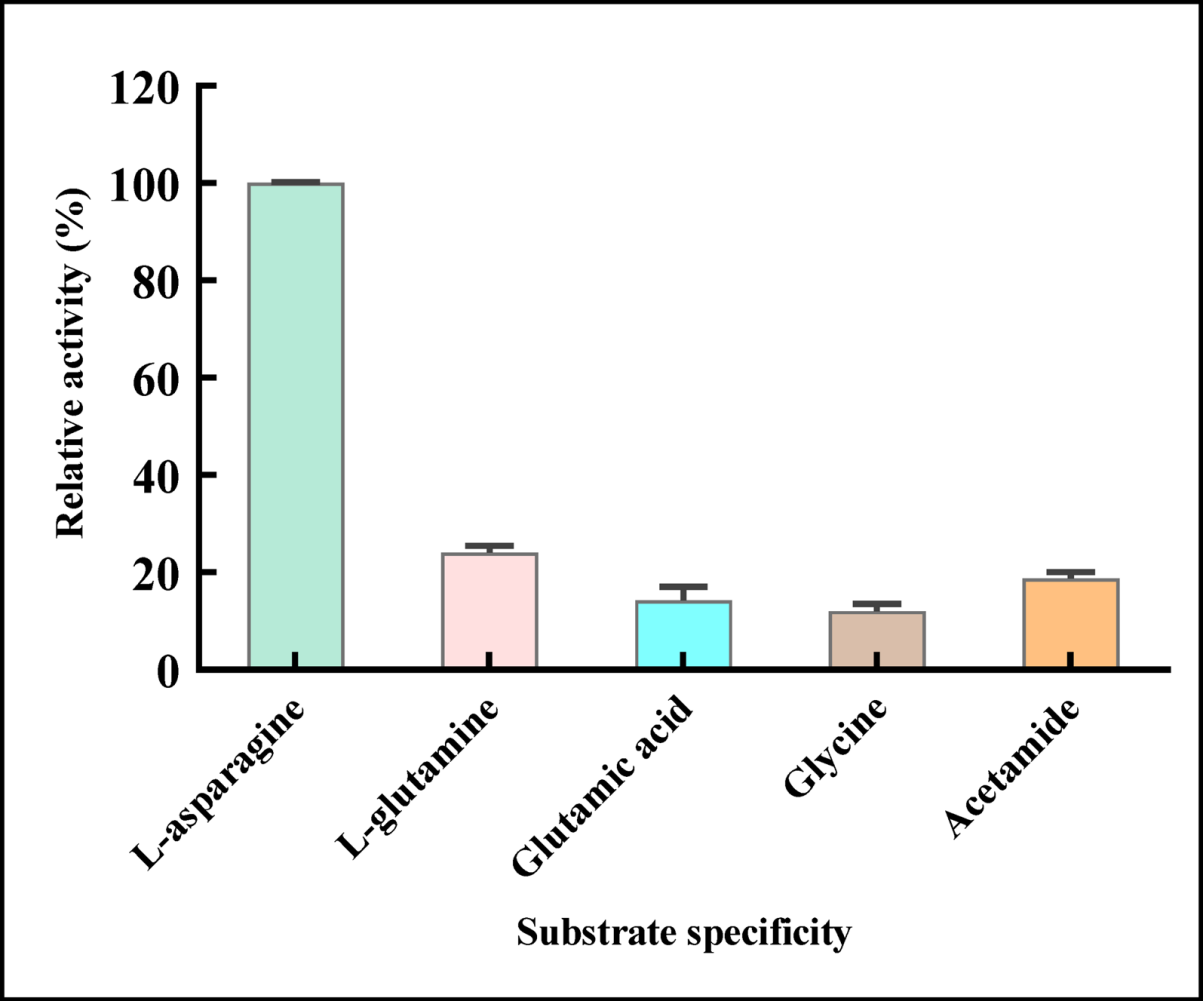
Effect of different substrates on the purified L-ASNase relative activity. The results were illustrated as means ± SD, where the F value indicated a very highly significant difference (F = 1016.0, *P* ≤ 0.0001)

 Figure 11 illustrated the effect of different substrates on the isolated L-ASNase catalytic activity. The enzyme demonstrated the highest specificity, exhibiting selectivity toward L-asparagine, at which L-ASNase showed a relatively higher hydrolytic rate with relative activity 100%. In contrast, specificity (23.97%) was observed for L-glutamine, while a lack of enzymatic activity was found using L-glutamic acid and glycine. The data of the current study indicated that the enzyme isolated from *P*. *ostreatus* was highly selective for its natural substrate L-asparagine. The results were in line with Kumar Meghavarnam and Janakiraman [96] from *F. culmorum* ASP-87. Also [109] reported similar observations, except that the enzyme showed minimal activity toward other compounds, including succinamic acid, D-asparagine, D-glutamine, D-aspartic acid, L-asparagine-t-butyl ester HCl, and N-acetyl-L-asparagine. Likewise, the enzyme from *M. hiemalis* and *Penicillium* sp. exhibited high substrate specificity, showing no activity against other tested amino acids, including L-glutamine, L-aspartic acid, L-arginine, L-histidine, and L-phenylalanine, and (L-aspartic acid, L-phenylalanine, L-glutamine, and L-histidine) [83], and [42], respectively.

#### Effect of storage times

**Fig. 12:**
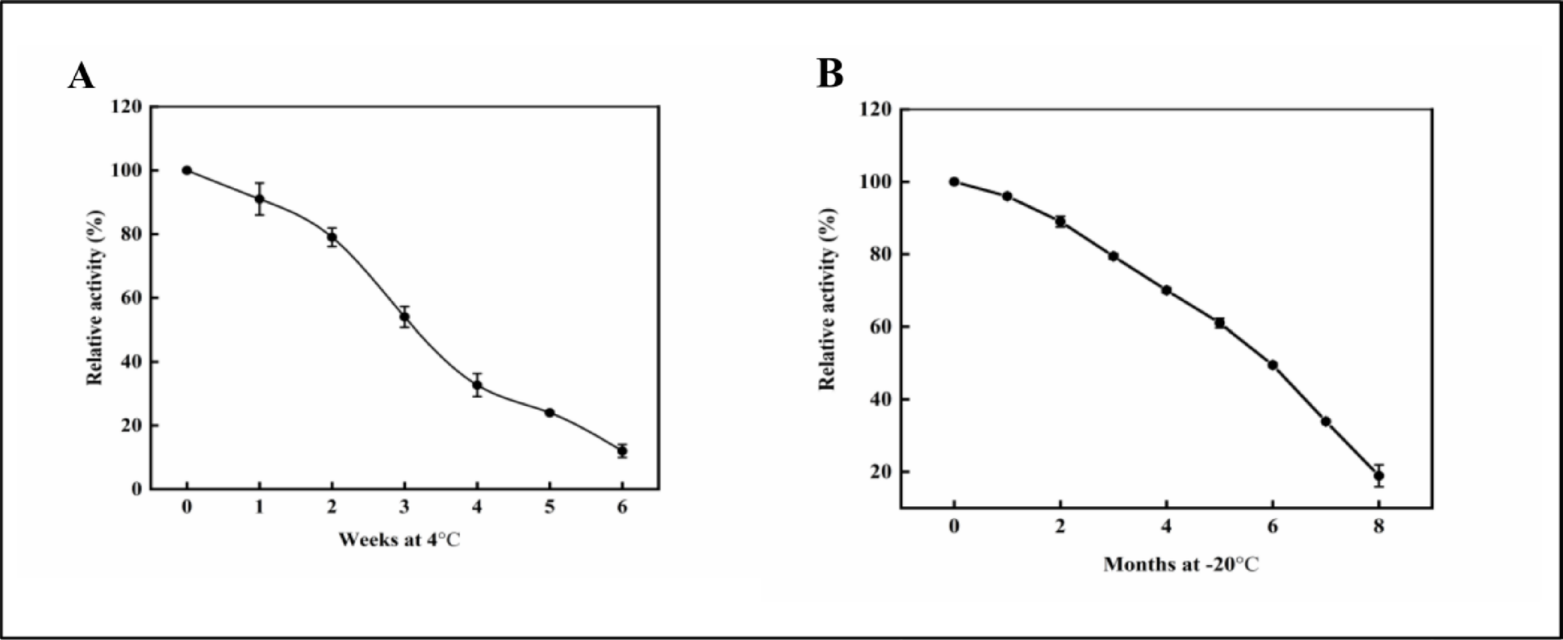
Effect of different storage times on the purified L-ASNase relative activity. **A**: At 4 °C. **B**: At – 20 °C. The results were illustrated as means ± SD, where the F value indicated a very highly significant difference (*P* ≤ 0.0001)

The results in Fig. 12A indicated that at 4 °C, L-ASNase enzyme gradually lost roughly 46% of its activity within 3 weeks of storage, while freezing at −20 °C, L-ASNase enzyme lost 50.5% of its activity within 6 months of storage and completely lost its activity within 8 months (Fig. 12B). According to [111] reported that L-ASNase from *Saccharomyces cerevisiae* ASP3 gene after 22 months of storage at −18 °C The crude asparaginase extract’s activity didn’t change. After five days of storage at 2 °C, less than 10% of the activity was retained. Further, [43] showed that, in all three storage temperatures, PEG-asparaginase maintained 95% of its initial enzymatic activity at 4 °C, −20 °C, and −80 °C for at least 14 days. Conversely, according to [112], *E. coli* L-ASNase maintained its activity for a minimum of seven days if appropriate sterile manipulation measures were followed at 8 °C.

### kinetic parameters

**Fig. 13:**
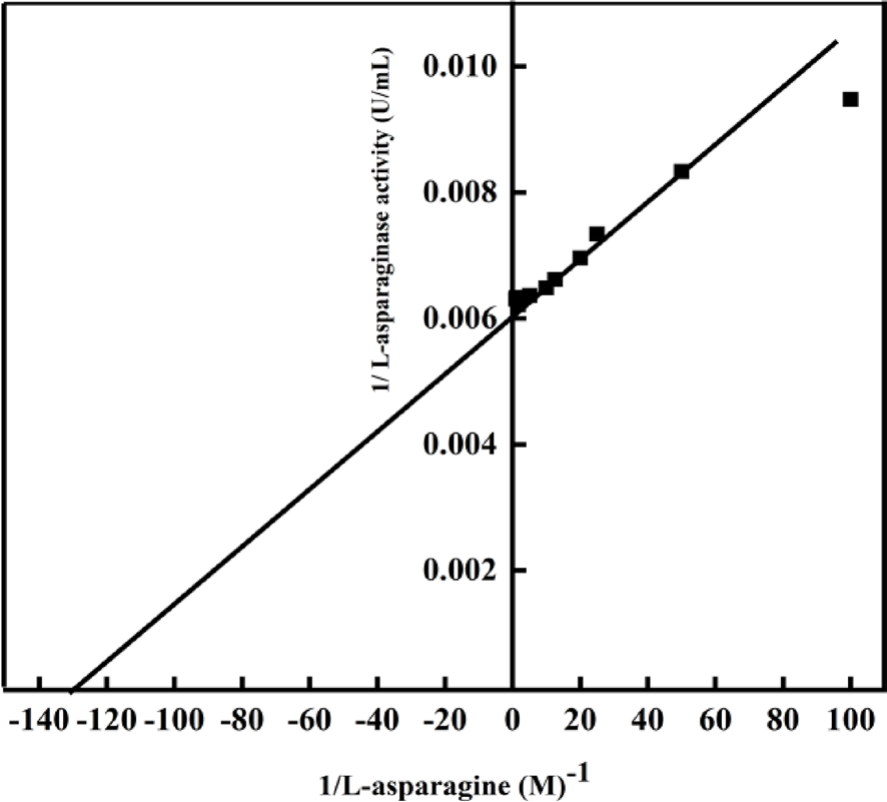
Lineweaver-Burk plots for the purified L-ASNase

The maximal velocity (*V*_*max*_) and Michael’s constant [113] of pure L-ASNase enzyme were ascertained with the aid of the Lineweaver-Burk plot. The *K*_*m*_ and *V*_*max*_ were found to be 7.7 mM and 167.78 U/mL, respectively, as in Fig. 13. The low *K*_*m*_ value indicated that the purified asparaginase had a strong affinity for the substrate asparagine. Similarly, [57] reported that *A. fumigates* W L002 L-ASNase had *K*_*m*_ and *V*_*max*_, 7.02 mM and 3553 µmol/ml/min, respectively. According to [40] observed that *L. theobromae* L-ASNase exhibited a *V*_*max*_ of 127.00 U/min and a *K*_*m*_ value of 9.37 µM. [87] observed that *A. oryzae* LBA 01 L-ASNase had a *K*_*m*_ value of 5.07 mM and a *V*_*max*_ of 57.14 U/mL. In contrast, AL Yousef [3] reported that *K*_*m*_ and *V*_*max*_ for the enzyme were 31.0 mM and 454 U/mL for *Fusarium* sp. While a slightly higher *K*_*m*_ (31.5 and 12.5 mM) and a *V*_*max*_ of 104.16 and 500 U/mL were exhibited by [41] and [99], respectively. On the other hand, Khalil et al. [100] and [114] showed that L-ASNase from *P. crustosum* and *A. oryzae IOC 3999* had a *K*_*m*_ constant value of (3.97 mM and 3.28 mM/L), and *V*_*max*_ (499.8 µmol/min/mg and 45.04 U/mL), respectively.

## Biological activity of pure L-ASNase from *P. ostreatus*

### Therapeutic applications

#### Antioxidant activity

**Fig. 14:**
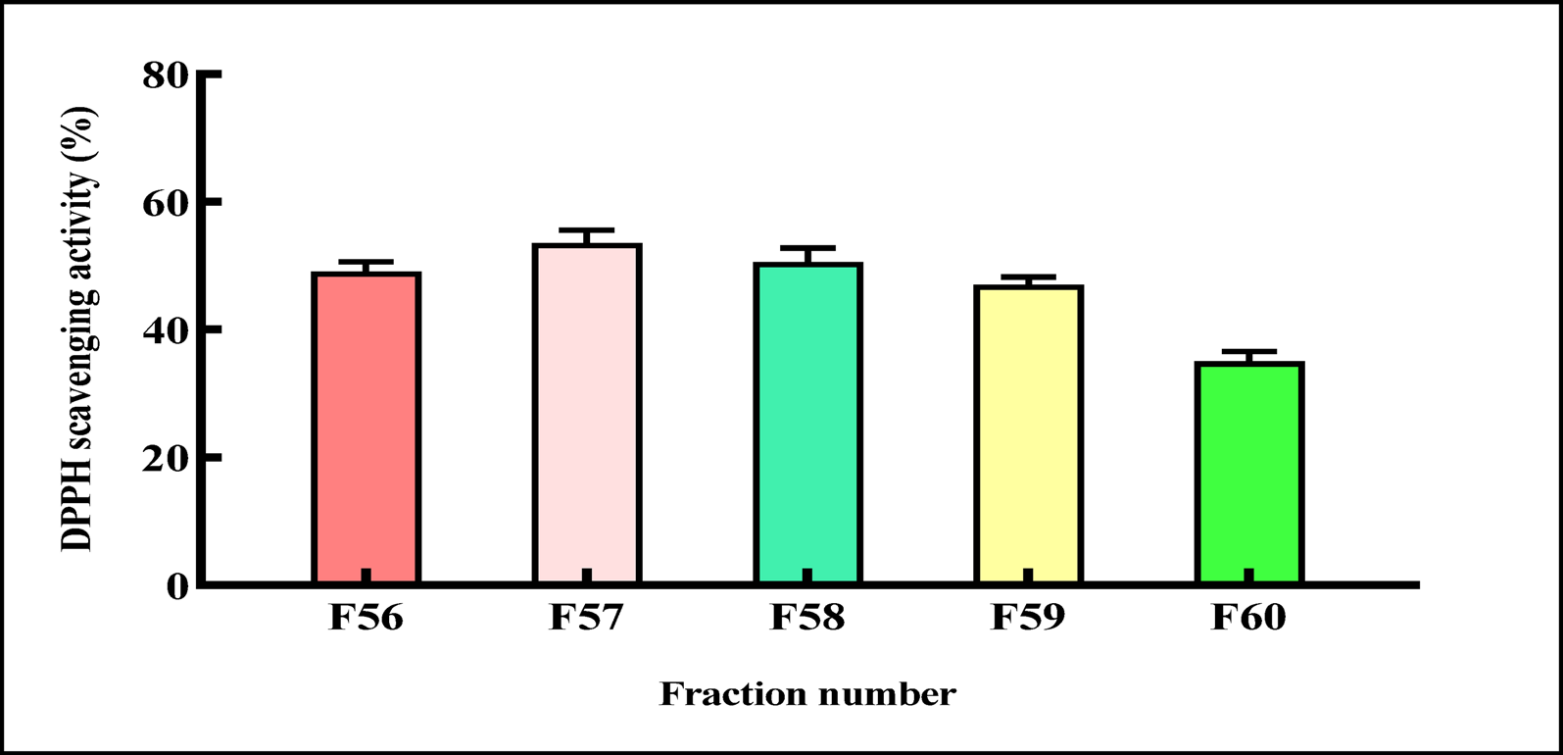
DPPH radical scavenging activity of the purified L-ASNase fractions (f56-f60). The results were illustrated as means ± SD

**Fig. 15:**
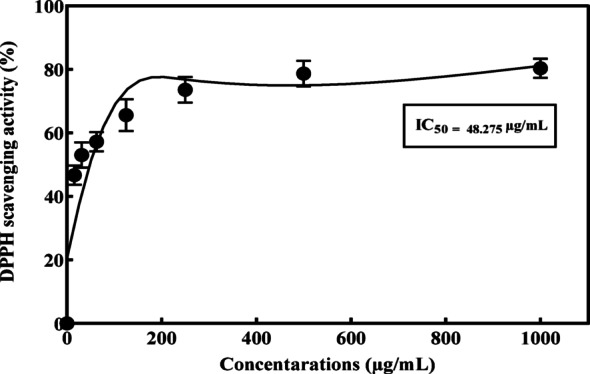
DPPH radical scavenging activity of serial dilutions from the purified L-ASNase fraction no. 57. The results were illustrated as means ± SD

The DPPH is a chromophore, a non-reactive radical cation colored purple. When an element or compound is combined with a DPPH solution that can give a hydrogen atom or an electron, such as water, it gives rise to the reduced form with the loss of its original purple color and turns to a pale yellow. According to these findings, *P. ostreatus* L-ASNase exhibited potent dose-dependent DPPH radical scavenging action, displaying an IC_50_ value at 48.275 µg/mL (Figs. 14 and 15). On the other hand, isolated L-ASNase from *F. equiseti* AHMF4 showed encouraging DPPH radical scavenging efficacy with an 69.12 IC_50_ µg/mL via El-Gendy et al. [39]. Also, fungal strains, including AYA 20 − 1 fusant, *A. fumigatus*, and mangrove *Avicennia germinans Aspergillus* sp., showed commendable dose-dependent antioxidant and scavenging activities with L-ASNase [110, 115, 116].

#### Anti-inflammatory activity

**Fig. 16:**
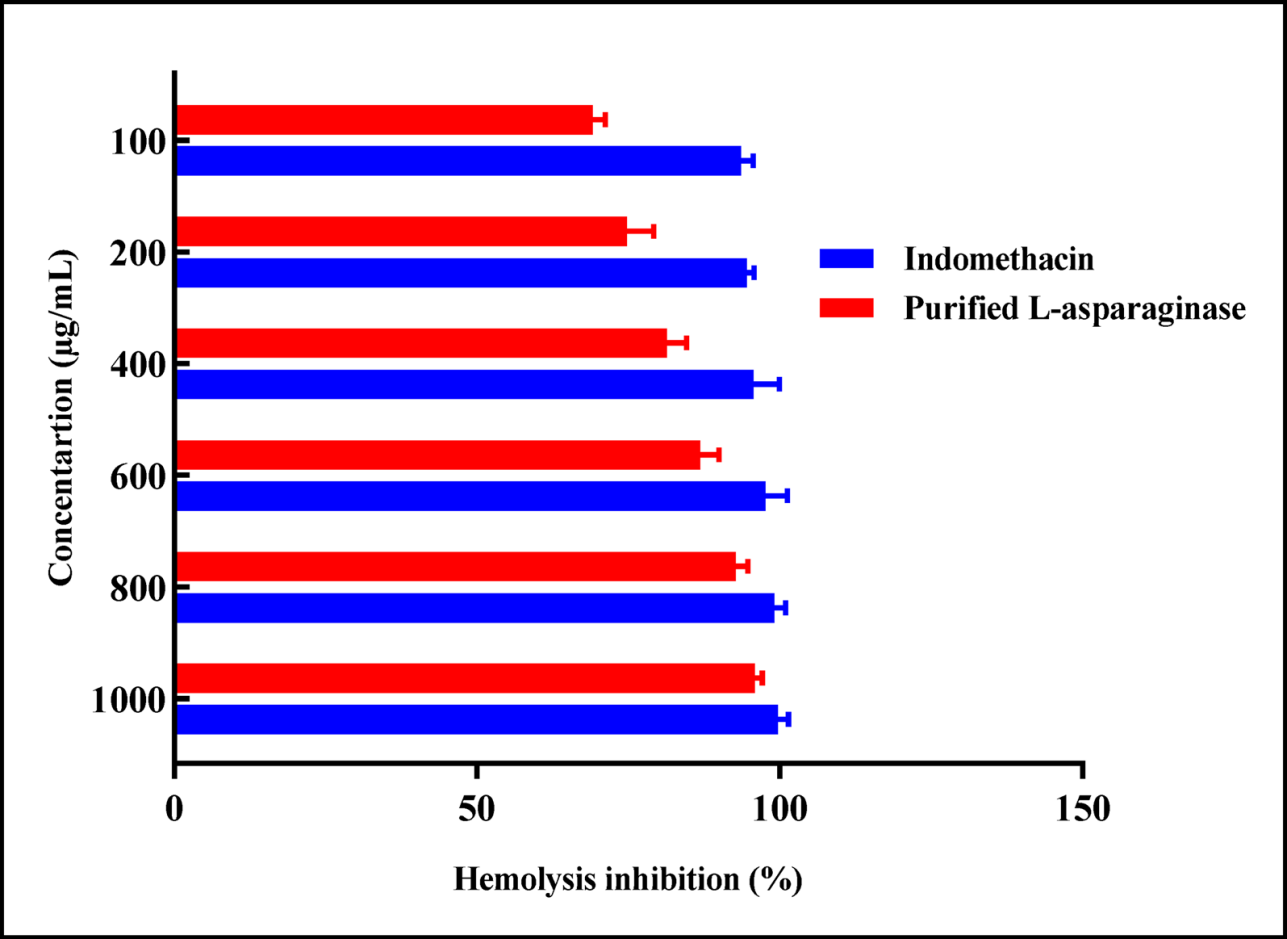
Anti-inflammatory activity of the purified L-ASNase via hemolysis inhibition measurement. The results were illustrated as means ± SD

Similar to the positive control medication indomethacin, it was found that the hemolysis inhibition percentage increases as the concentration of pure L-ASNase increases. Hemolysis inhibition % was 69.1, 74.7, 81.4, 86.9, 92.7, and 95.9% utilizing pure L-ASNase, while it was 93.6, 94.6, 95.7, 97.7, 99.1, and 99.7% compared to standard indomethacin, respectively. Interestingly, from the results obtained in Fig. 16, L-ASNase has an anti-inflammatory effect in a dose-dependent manner. Since RBC membranes resemble lysosome membranes, the mechanism of L-ASNase ‘s anti-inflammatory activity was measured by the suppression of hypotonicity-induced RBC membrane lysis [117]. The hemolytic action of the hypotonic solution is caused by an excess fluid buildup inside the cell, which causes the cell membrane to burst. Red cell membrane (RCM) damage increases the cell’s sensitivity to further harm from radical-induced peroxidation of lipids [118]. Lysosomes lyse and release their constituent enzymes during inflammatory episodes, leading to a range of diseases. Nonsteroidal anti-inflammatory medications (NSAIDs) function by either stabilizing lysosomal membranes or preventing the release of lysosomal enzymes [117]. RBCs’ membranes lyse in response to toxic molecules, including heat, methyl salicylate, hypotonic medium, or phenylhydrazine, which causes hemolysis and hemoglobin oxidation [119]. According to Mahabal and Kaliwal [120] L-ASNase isolated from *A. tamarii* showed dose-dependent inhibition of important inflammatory processes in vitro, including over 50% protection against hemolysis and hypotonic-induced erythrocyte lysis. Similarly, L-ASNase from *F. solani* and *Solanum nigrum* showed a significant anti-inflammatory impact [101, 121]. Additionally, mechanistic analysis by Song et al. [122] showed that cytokines promote inflammation, can be inhibited by L-ASNase, including TNF-α and IL-6, in activated macrophages, suggesting that it may be able to modify inflammatory responses at the molecular level.

#### Anti-tumor activity

**Fig. 17:**
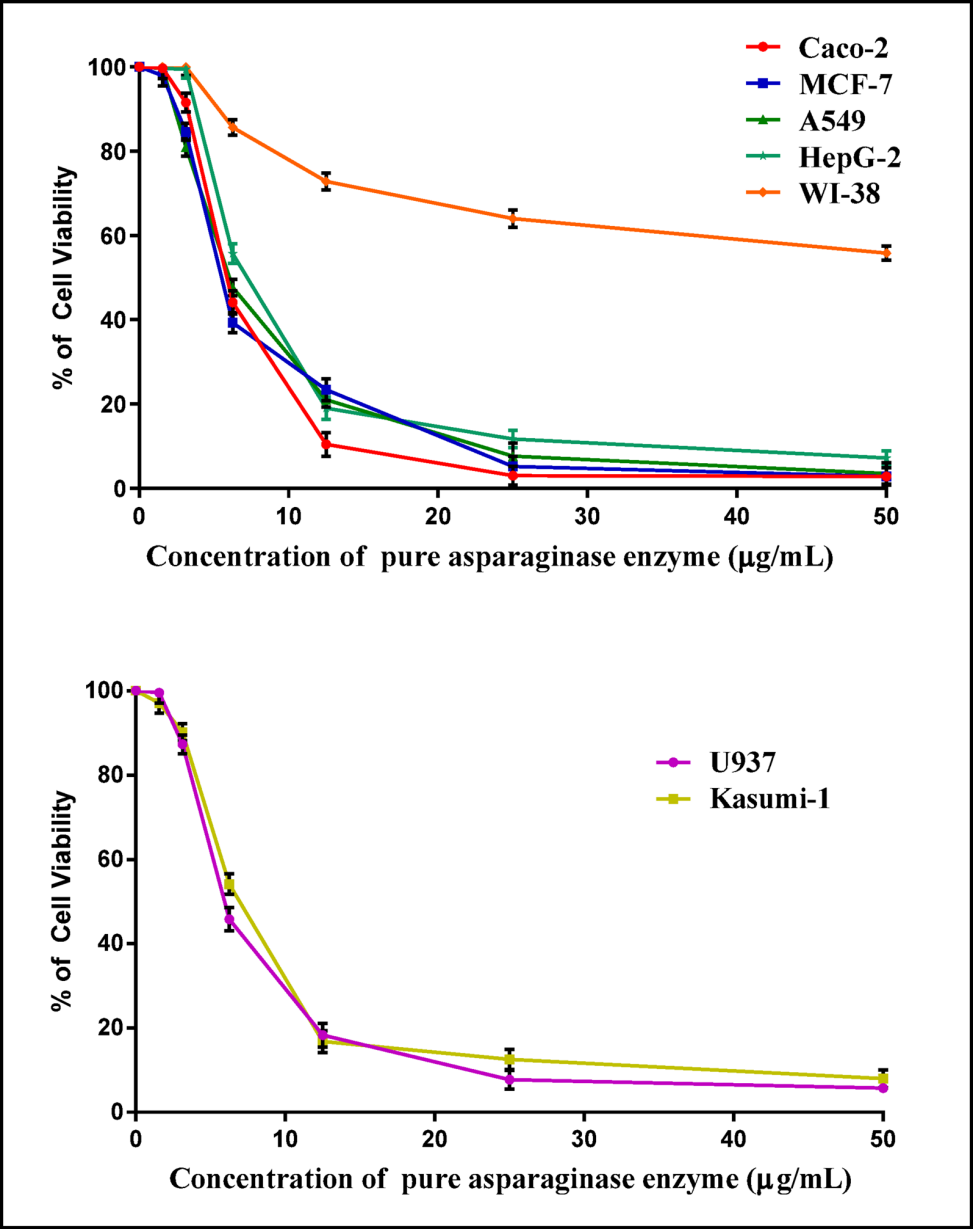
Photographs illustrate the effect of the purified L-ASNase on the growth inhibition of six human tumor cell lines, hepatocellular carcinoma cell line (HepG-2), breast carcinoma cell line (MCF-7), colon carcinoma (Caco-2), lung cells (A549), acute myoblastic leukemia (Kasumi-1), histiocytic lymphoma (U937), and normal cells (WI-38). The results were illustrated as means ± SD

The results obtained in Fig. 17 showed that the purified L-ASNase exhibited various degrees of anti-tumor effect towards the panel of cancer cell lines. The highest inhibitory effect of L-ASNase was recorded towards Colon carcinoma (Caco-2) at 97.2% with an IC_50_ equal to 5.49 ± 0.03 µg/mL, followed by the breast cell line (MCF-7) at 97.2% with an IC_50_ equal to 5.86 ± 0.08 µg/mL. The least inhibitory effect of L-ASNase was obtained towards acute myoblastic leukemia (Kasumi-1) at 92.0% with an IC_50_ equal to 7.76 ± 0.15 µg/mL. Interestingly, L-ASNase showed a selective safety profile by showing minimal adverse effects on non-cancerous human cells. According to Moharib [123], L-ASNase’s anticancer action may be related to its ability to inhibit the development of cancerous cell lines, which may stop the cell cycle and cause apoptosis. L-ASNases and associated enzymes could be useful medicinal agents in the treatment of malignancies, according to recent medical research on cancer cells. This suggests that these protein molecules show significant promise for future clinical applications [67]. In contrast to tumor cells, normal cells can use asparagine synthetase to produce intracellular L-asparagine in the event of a deficit. For appropriate growth, neoplastic cells, on the other hand, need a sufficient foreign supply of L-asparagine because they lack this enzyme. When there is no such source of L-asparagine, the levels are quickly exhausted, which causes the cancerous cells to die. The synthesis of DNA, RNA, and proteins is inhibited when L-ASNase depletes the plasma of L-asparagine, causing apoptosis. Therefore, L-ASNase ‘s therapeutic action is the reduction of these amino acids in lymphatic tumor cells, which results in the cells’ malnutrition and eventual destruction [67, 124]. L-ASNase is recognized as a cytotoxic agent against acute lymphoblastic leukemia in the most recent edition of the WHO Essential Medicines List [125]. The current study was in line with El-Naggar et al. [126], which investigated *Streptomyces fradiae* NEAE-82 L-ASNase ‘s efficiency against Caco-2, and HepG-2 cancer cells and found its IC_50_ was between 2 and 4 µg/mL. *A. fumigatus* L-ASNase showed strong inhibitory effects on breast cancer cells (MDA-MB-231), resulting in 71%, 87.7%, and 96.5% cell death at concentrations of 5, 10, and 20 µg/mL, respectively, as reported by Benchamin et al. [115]. *A. oryzae* CCT 3940 isolated L-ASNase exhibited potent anti-proliferative effects against several tumor-derived cell cultures, such as PC-3 (prostate), NCI-ADR/RES (ovary), NCI-H40 (lung), UACC-62 (melanoma), 786-0 (kidney), and K562 (leukemia) cells [87]. Additionally, Othman et al. [67] found that the IC_50_ values were 28.6 (A549), 72.4 (MCF-7), 23.6 (HCT), and 23.4 (HepG-2) µg/mL by *R. oryzae* AM16 L-ASNase.Moreover, inhibition in the proliferation of Hela, Hep-2, HepG-2and HCT-116was seen upon treatment with 10, 20, 60, and 80 µg/mL of *F. equiseti* AHMF4 L-ASNase, with corresponding IC_50_ values of 2.0, 5.0, 12.40, and 8.26 µg/mL [39]. Additionally, MCF-7 breast cancer cells showed greater resistance to the cytotoxic consequences of L-ASNase treatment possessing a 22.8 µg/mL IC_50_, following L-ASNase treatment, respectively, El**-**Gendy et al. [39]. Additionally, Hassan et al. [41] mentioned that *A. terreus* L-ASNase demonstrated antineoplastic activity against HepG-2, MCF-7, and HCT-116 cells, with an IC_50_ between 3.79 and 12.6 µg/mL. On the contrary, a study by Al Yousef [3] reported that when *Fusarium* sp. L-ASNase was investigated for its cancer-fighting capability on RAW2674 leukemic cell lines; it demonstrated anti-leukemia potency with an IC_50_ of 50.1 µg/mL.

### Food application

#### L-ASNase as a food processing aid

**Fig. 18:**
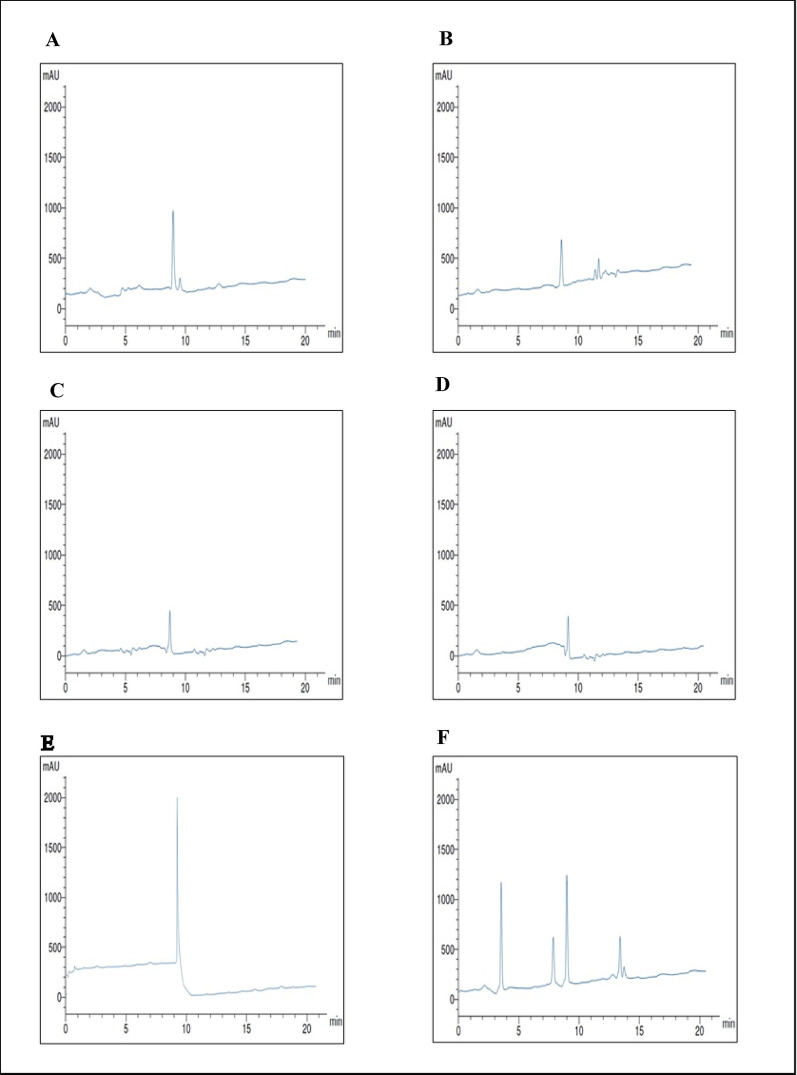
HPLC charts of the potato sample treated with the purified L-ASNase at different times. **A**: For 30 min. **B**: for 60 min. **C**: for 90 min. **D**: for 120 min. **E**: HPLC chart of standard acrylamide sample. **F**: HPLC chart of the untreated fried potato sample

L-ASNase represents one of the substitute methods for mitigating acrylamide in food products. In the current study, HPLC was employed to identify the decline of acrylamide in fried potatoes. The results demonstrated a significant mitigation of acrylamide in potato samples treated with the purified L-ASNase over different periods (30, 60, 90, and 120 min) following 6–7 min of heating to 180 °C. In potato samples, the acrylamide concentration decreased as L-ASNase treatment time increased, with the enzyme administered at 60.885 U/mL. The optimum concentration of acrylamide was formed after treatment of the potato sample with the purified L-ASNase for 30 min, with a retention time of 9.0 min and a concentration of 10.11 µg/g (Fig. 18 A), followed by treatment of the potato sample for 60 min with a retention time of 8.8 min. Its concentration was 6.23 µg/g (Fig. 18B), followed by treatment of the potato sample for 90 min with a retention time of 9.1 min. Its concentration was 4.02 µg/g (Fig. 18 C). And the lowest concentration formed after treatment of the potato sample with the purified L-ASNase for 120 min, with a retention time of 9.1 min and its concentration was 2.35 µg/g (Fig. 18D), compared to the untreated potato sample (control sample), which was detected with a retention time of 9.0 min with 22.65 µg/g (Fig. 18E). Standard acrylamide was detected with a retention time of 9.0 min and 45.0 µg/g (Fig. 18 F). To assess the impact of *Acinetobacter soli* L-ASNase, [105] measured the levels of L-asparagine in pre-processed potato slices and acrylamide produced during thermal processing in fried potato snacks via liquid chromatography combined with tandem mass spectrometry (LC-MS/MS). Following enzymatic treatment, potatoes’ free L-asparagine content dropped by 22.0%, from 64.6 ± 1.0 mg/kg to 50.4 ± 0.7 mg/kg. The control group had an acrylamide content of 0.446 ± 0.011 mg/kg. After samples were treated with 30.0 U/mL of the pure L-ASNase, the amount of acrylamide was 0.197 mg/kg, suggesting its level dropped by roughly 55.9%. According to Mohan Kumar and Manonmani [106], the amount of acrylamide in blanched potato snacks that were not treated with an enzyme was 173.51 ± 0.8 µg/g. In contrast, the amount of potato fries that were treated with L-ASNase was 7.62 ± 0.5 µg/g. This suggested that L-asparagine was converted to L-aspartic acid and ammonia, which stopped the Millard reaction derivatives as acrylamide content in baked products like potatoes from forming. The significance of L-ASNase is used not only in the pharmaceutical and healthcare sectors but also in the food industry to address issues related to the undesired synthesis of acrylamide, a neurotoxic and carcinogenic compound found in some meals, including potato chips and all foods high in carbohydrates [127].

### GC-MS analysis

**Fig. 19:**
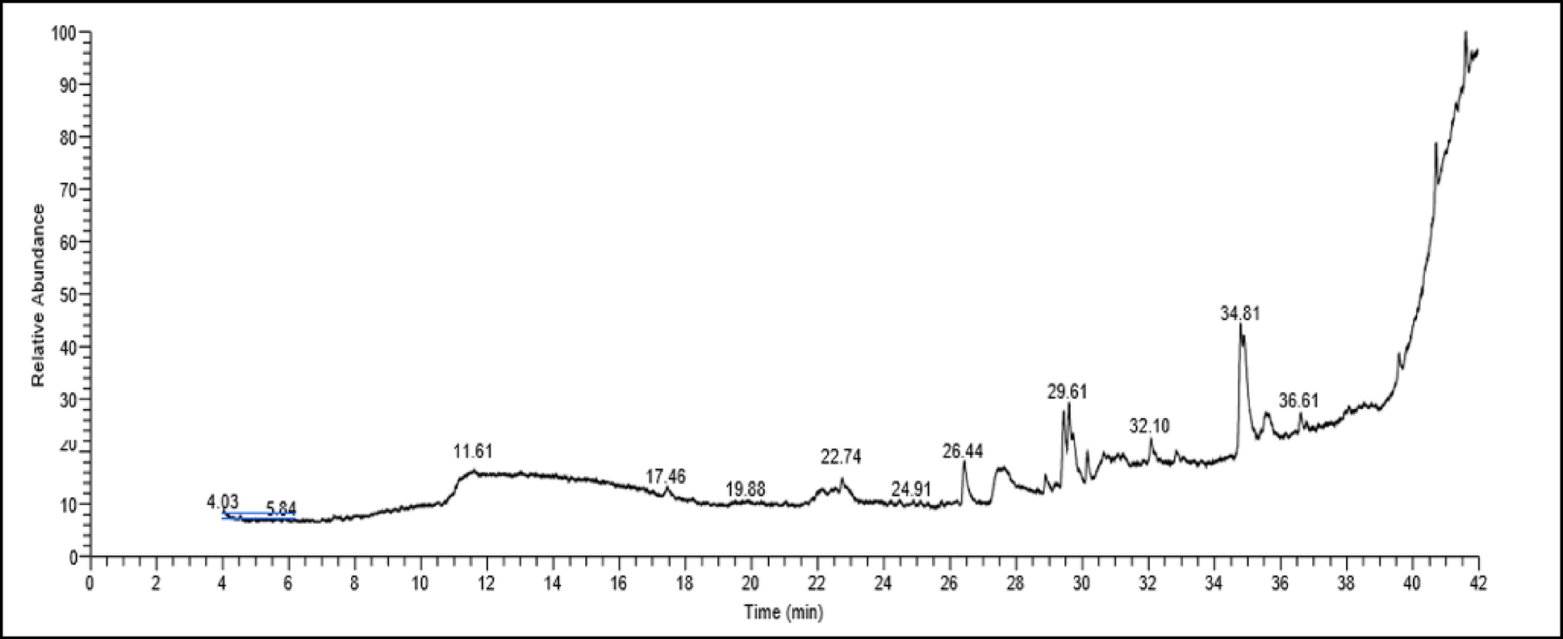
GC-MS chromatogram of the control potato sample

**Fig. 20:**
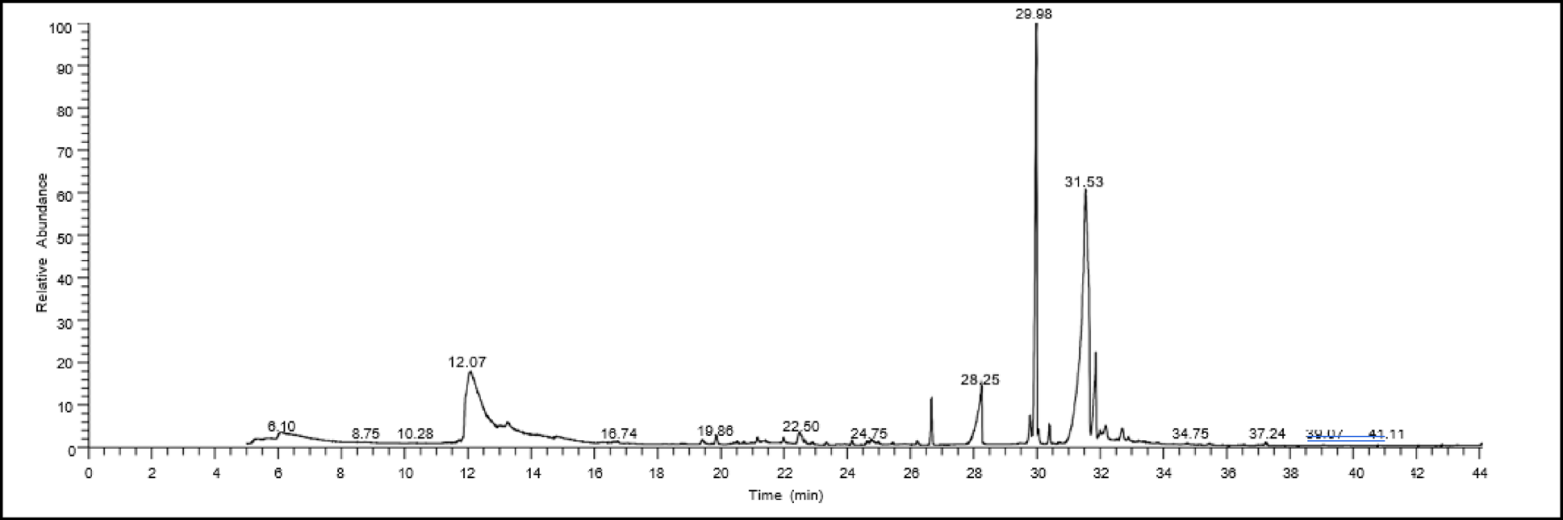
GC-MS chromatogram of the purified L-ASNase-treated sample for 120 min

The GC-MS examination of acrylamide control and the purified L-ASNase-treated samples after 120 min revealed significant variations in their chemical profiles (Figs. 19 and 20). Following enzymatic treatment, the total compounds still present in the purified L-ASNase-treated sample were 1,2-BENZENEDIOL, 2-Cyclohexen-1-one, 4-(3-hydroxybutyl)−3,5,5-trimethyl-, Benzene, (1-methyldodecyl)-, 9,12-OCTADECADIENOIC ACID, OCTADECANOIC ACID, Methyl ester, 5-Benzofuranacetic acid, 6-ethenyl-2,4,5,6,7,7a-hexahydro-3,6-dimethyl-à-methylene-2-oxo-, methyl ester, as shown in Table 2. These compounds exhibited biological significance for human health and are presented along with their potential properties and functions, which are in line with the previous studies [128–132].

**Table 2:** GC-MS analysis for determining the bioactive compounds in untreated and treated potato samples with purified L-ASNase

	Control sample			Treated sample		
	RT	Compound name	Peak area %	RT	Compound name	Peak area %
1	11.18	9,12,15-OCTADECATRIENOIC ACID, (2-PHENYL-1,3-DIOXOLAN-4-YL)METHYL ESTER	2.36	6.06	4-Heptenal, (Z)-	0.75
2	11.23	9,12,15-OCTADECATRIENOIC ACID, (2-PHENYL-1,3-DIOXOLAN-4-YL)METHYL ESTER	0.75	12.04	1,2-BENZENEDIOL	13.10
3	11.33	8-AZABICYCLO[3.2.1]OCTAN-3-OL, 8-METHYL-, BENZOATE (ESTER), EXO-	1.41	13.25	1,2-BENZENEDIOL	0.53
	11.47	9,12,15-OCTADECATRIENOIC ACID, (2-PHENYL-1,3-DIOXOLAN-4-YL)METHYL ESTER	2.45	19.42	2(3 H)-FURANONE, 5-HEPTYLDIHYDRO-	0.42
5	11.54	9,12,15-OCTADECATRIENOIC ACID, (2-PHENYL-1,3-DIOXOLAN-4-YL)METHYL ESTER	1.16	19.86	1,2-BENZENEDICARBOXYLIC ACID, DIETHYL ESTER	0.70
6	11.61	8-AZABICYCLOOCTANE-2-CARBOXYLIC ACID, 3-(BENZOYLOXY)−8-METHYL-, METHYL ESTER	2.85	21.16	12,15-OCTADECADIYNOIC ACID, METHYL ESTER	0.35
7	11.79	9,12,15-OCTADECATRIENOIC ACID, (2-PHENYL-1,3-DIOXOLAN-4-YL)METHYL ESTER	1.27	21.99	Benzene, (1-methyldecyl)-	0.36
8	11.88	9,12,15-OCTADECATRIENOIC ACID, (2-PHENYL-1,3-DIOXOLAN-4-YL)METHYL ESTER	0.87	22.49	2-Cyclohexen-1-one, 4-(3-hydroxybutyl)−3,5,5-trimethyl-	1.41
9	11.93	9,12,15-OCTADECATRIENOIC ACID, (2-PHENYL-1,3-DIOXOLAN-4-YL)METHYL ESTER	0.65	24.15	Benzene, (1-methylundecyl)-	0.29
10	11.98	Glucosamine, N-acetyl-N-benzoyl-	0.82	26.22	Benzene, (1-methyldodecyl)-	0.31
11	12.04	9,12,15-OCTADECATRIENOIC ACID, (2-PHENYL-1,3-DIOXOLAN-4-YL)METHYL ESTER	0.70	26.66	Hexadecanoic acid, methyl ester	2.63
12	12.10	9,12,15-OCTADECATRIENOIC ACID, (2-PHENYL-1,3-DIOXOLAN-4-YL)METHYL ESTER	0.71	28.24	n-Hexadecanoic acid	5.32
13	12.17	9,12,15-OCTADECATRIENOIC ACID, (2-PHENYL-1,3-DIOXOLAN-4-YL)METHYL ESTER	2.10	29.78	9,12-OCTADECADIENOIC ACID, METHYLESTER, (E, E)-	1.60
14	12.33	5-Amino-1-benzoyl-1 H-pyrazole-3,4-dicarbonitrile	0.76	29.97	9-Octadecenoic acid, methyl ester, (E)-	28.62
15	12.37	8-AZABICYCLO[3.2.1]OCTANE-2-CARBOXYLIC ACID, 3-(BENZOYLOXY)−8-METHYL-, METHYL ESTER	0.46	30.05	11-Octadecenoic acid, methyl ester	0.24
16	22.75	Ascaridole epoxide	2.35	30.39	OCTADECANOIC ACID, METHYL ESTER	1.16
17	26.44	PENTADECANOIC ACID, 14-METHYL-, METHYL ESTER	4.72	31.54	trans-13-Octadecenoic acid	31.73
18	27.46	Dasycarpidan-1-methanol, acetate (ester)	2.94	31.86	OCTADECANOIC ACID	6.89
19	27.50	HEXADECANOIC ACID, 2,3-DIHYDROXYPROPYL ESTER	0.83	32.00	5-Benzofuranacetic acid, 6-ethenyl-2,4,5,6,7,7a-hexahydro-3,6-dimethyl-à-methylene-2-oxo-, methyl ester	0.66
20	27.55	Estra-1,3,5(10)-trien-17á-ol	1.51	32.17	HI-OLEIC SAFFLOWER OIL	1.75
21	27.63	Estra-1,3,5(10)-trien-17á-ol	5.25	32.70	[1,1’-Bicyclopropyl]−2-octanoic acid, 2’-hexyl-, methyl ester	1.21
22	29.44	HEXADECADIENOIC ACID, METHYL ESTER	5.35			
23	29.61	9,12-Octadecadienoyl chloride, (Z, Z)	5.35			
24	29.73	9,12-Octadecadienoyl chloride, (Z, Z)	5.18			
25	30.17	METHYL-9,9,10,10-D4-OCTADECANOATE	1.99			
26	30.65	9-OCTADECENOIC ACID (Z)-	2.48			
27	30.78	9-OCTADECENOIC ACID (Z)-	0.91			
28	30.83	9-OCTADECENOIC ACID (Z)-	0.99			
29	30.91	9-OCTADECENOIC ACID (Z)-	1.13			
30	31.09	9-OCTADECENOIC ACID (Z)-	2.33			
31	31.22	9-OCTADECENOIC ACID (Z)-	2.90			
32	32.09	9-OCTADECENOIC ACID (Z)-	2.91			
33	34.80	9,12-Octadecadienoic acid (Z, Z)-, 2-hydroxy-1-(hydroxymethyl)ethyl ester	8.39			
34	34.91	9,12-Octadecadienoyl chloride, (Z, Z)-	13.04			
35	35.21	HEXADECADIENOIC ACID, METHYL ESTER	1.38			
36	35.58	1-Heptatriacotanol	2.88			
37	35.67	9,12-Octadecadienoyl chloride, (Z, Z)	2.79			
38	36.62	ISOCHIAPIN B	1.75			
39	39.60	ISOCHIAPIN B	1.35			

Furthermore, several compounds found in the acrylamide control were absent from the treated L-ASNase sample, which may indicate that L-ASNase has initiated enzymatic hydrolysis, transformation, or metabolic conversion processes.

The total compounds that disappeared from the treated L-ASNase sample were 9, 12, 15-Octadecatrienoic acid, (2-Phenyl-1, 3-dioxolan-4-yl) methyl ester, 8-Azabicyclooctan-3-ol, Benzoate (ester), Exo-, 8-azabicyclooctane-2-carboxylic acid, 3-(Benzoyloxy)−8-Methyl Glucosamine, N-acetyl-N-benzoyl, 5-Amino-1-benzoyl-1 H-pyrazole-3, 4-dicarbonitrile, Dasycarpidan-1-methanol, acetate (ester), Hexadecanoic acid, 2, 3-dihydroxypropyl ester, Estra-1, 3, 5(10)-trien-17á-ol,, 9, 12-Octadecadienoyl chloride, (Z, Z), 9-octadecenoic acid, 2-hydroxy-1-(hydroxymethyl)ethyl ester, 1-Heptatriacotanol, Isochiapin b. These major compounds elucidated varying toxic effects, which was confirmed with the previous studies [133–135].

## Conclusion

The current study reveals that *P. ostreatus* AUMC 16,015 is an excellent source of high-potential L-ASNase enzyme grown on agricultural wastes in various physiological processes. Also, it has a promising antioxidant, anti-inflammatory, antitumor, and food safety impact. Therefore, *P. ostreatus* AUMC 16,015 L-ASNase was recommended to be used as an excellent source in many biomedical and food applications.

## Limitations of the study and future perspectives

Future research will focus on scaling up solid-state fermentation using cost-effective bioreactors and exploring genetic or metabolic engineering to boost fungal enzyme yield. Importantly, in vivo studies and further clinical evaluations are required to validate the enzyme’s therapeutic efficacy, particularly its antileukemic and anti-inflammatory properties. Further optimization will include screening new agro-wastes and investigating synergistic effects with other chemicals. Ultimately, incorporating advancements in downstream processing is essential to creating sustainable and commercially viable L-ASNase production platforms for pharmaceutical and food industry applications.

## Data Availability

No datasets were generated or analysed during the current study.

## Abbreviations

IU: International unit
U/gds: Unit per gram of initial dry solid substrate
µg: Micro gram
Mg: Milli gram
MCD: Modified czapex dox
PDA: Potato dextrose agar
BSA: Bovine serum albumin
SDS-PAGE: Sodium dodecyl sulphate polyacrylamide gel electrophoresis
EDTA: Ethylene diamine tetraacetic acid
DPPH: (2,2-diphenyl-1-picrylhydrazyl)
MTT: (3-(4,5-dimethylthiazol)-2,5-diphenyltetrazolium bromide)
HPLC: High-performance liquid chromatography
